# Oblivious network intrusion detection systems

**DOI:** 10.1038/s41598-023-48475-w

**Published:** 2023-12-15

**Authors:** Mahmoud AbdelHafeez Sayed, Mostafa Taha

**Affiliations:** Systems and Computer Engineering, Carleton University, 1125 Colonel By Dr, Ottawa, K1S 5B6 ON Canada

**Keywords:** Electrical and electronic engineering, Information technology

## Abstract

A main function of network intrusion detection systems (NIDSs) is to monitor network traffic and match it against rules. Oblivious NIDSs (O-NIDS) perform the same tasks of NIDSs but they use encrypted rules and produce encrypted results without being able to decrypt the rules or the results. Current implementations of O-NIDS suffer from slow searching speeds and/or lack of generality. In this paper, we present a generic approach to implement a privacy-preserving O-NIDS based on hybrid binary gates. We also present two resource-flexible algorithm bundles built upon the hybrid binary gates to perform the NIDS’s essential tasks of direct matching and range matching as a proof of concept. Our approach utilizes a Homomorphic Encryption (HE) layer in an abstract fashion, which makes it implementable by many HE schemes compared to the state-of-the-art where the underlying HE scheme is a core part of the approach. This feature allowed the use of already-existing HE libraries that utilize parallelization techniques in GPUs for faster performance. We achieved a rule encryption time as low as 0.012% of the state of the art with only 0.047% of its encrypted rule size. Also, we achieved a rule-matching speed that is almost 20,000 times faster than the state of the art.

## Introduction

Network intrusion detection system (NIDS) is a critical component of an enterprise targeted at protecting the internal network and information systems from malicious intruders  ^1^. Typically, a NIDS is composed of three components: a network sensor, an analysis server, and a management console. The database of all the known threats is represented as a list of rules or a ruleset. The analysis server of NIDS will scan the network traffic in/out of the network sensor while applying conditions stipulated in the ruleset to find any packet that has a high chance of being part of a malicious activity, raising an alert in the management console. In this research, we aim to design and build an efficient and scalable Oblivious NIDS (O-NIDS), where the ruleset is created and encrypted by trusted personnel, while the network sensor and analysis server operate at a remote, insecure environment hosted and administrated by an adversary who is untrusted, honest-but-curious observer (HCB). The analysis server operates on the encrypted ruleset to generate encrypted alerts, which are communicated back in a secure way to the trusted personnel on a trusted management console. Such technology allows providing NIDS as a cloud service and enables cybersecurity protection at remote hostile locations without revealing the secret ruleset which can be classified according to some government regulations or the trade secret of cybersecurity research companies. In these applications context, the main idea is to separate between the rules creator and evaluator. The rules creator is a trusted authority, e.g. governmental agency, which creates and obfuscates the rules, while the rules evaluator is a remote party, e.g. a cloud service, responsible for performing the matching process between the obfuscated rules and the plaintext data under inspection without being able to understand the matching results. This is different from the currently used cloud IDS technologies like Google Cloud IDS^2^ or Azure Cloud firewall^3^, where the matching/detection results exist in plain and a role-based access model is used to prevent unauthorized access to these results. In our proposed model, the results themselves are encrypted on the data level so the rules-evaluator side has essentially no capability to decrypt or understand the results.

To achieve the principle idea of keeping the rules and the results incomprehensible to the rules-evaluator, Homomorphic Encryption (HE) schemes can be utilized. HE is a cryptographic technology that allows performing computations on encrypted data without accessing the secret key  ^4^ and, consequently, without being able to decrypt the encrypted input or output data of such computations. While the available HE technology can provide a working prototype for the core problem, there is no efficient or scalable solution that can support a modern NIDS design with thousands of rules against multiple Gbits/s of network traffic.

### Main contributions

This journal manuscript is an extended version of a WIP paper in HOST2022, entitled: Mahmoud Abdelhafeez Sayed and Mostafa Taha. Oblivious Intrusion Detection System. In *2022 IEEE International Symposium on Hardware Oriented Security and Trust (HOST)*. IEEE, 2022. In this paper, we present a privacy-preserving NIDS by implementing hybrid matching operations on the very fundamental binary level. Developing the matching at a bit level allows natural extension to any binary circuit or Finite State Machine (FSM) and eventually to a complete oblivious NIDS. Moreover, we propose two algorithms built on top of the proposed hybrid operations that implement two fundamental NIDS tasks; direct matching and range matching. The proposed algorithms introduce wide resource-performance trade-offs to fit with the resources and performance requirements of different environments. In addition, the proposed hybrid gates approach is library-independent, i.e., they can be implemented with any homomorphic encryption library that supports binary or radix-2 operations. To demonstrate the advantage of library independence, we propose an implementation that utilizes a GPU-accelerated HE library to enhance the performance. The proposed solutions are tested using TFHE^5^, Microsoft SEAL^6^, and PALISADE^7^ on CPU and cuFHE^8^ on GPU. In a nutshell, compared to the state of the art, the proposed solution provides:

1. HE library independency.
2. Higher Security Level.
3. Faster rule encryption time.
4. Smaller encrypted rule size.
5. Lower memory requirements in most cases.
6. Faster matching time.
7. Flexible time $\times$ memory trade.
8. Faster decryption time
9. Modular structure that allows partial modifications and updates to the rules and also allows combining results of different modules/algorithms in a straightforward way.

These features will be discussed in detail in section “Discussion”.

**Figure 1:**
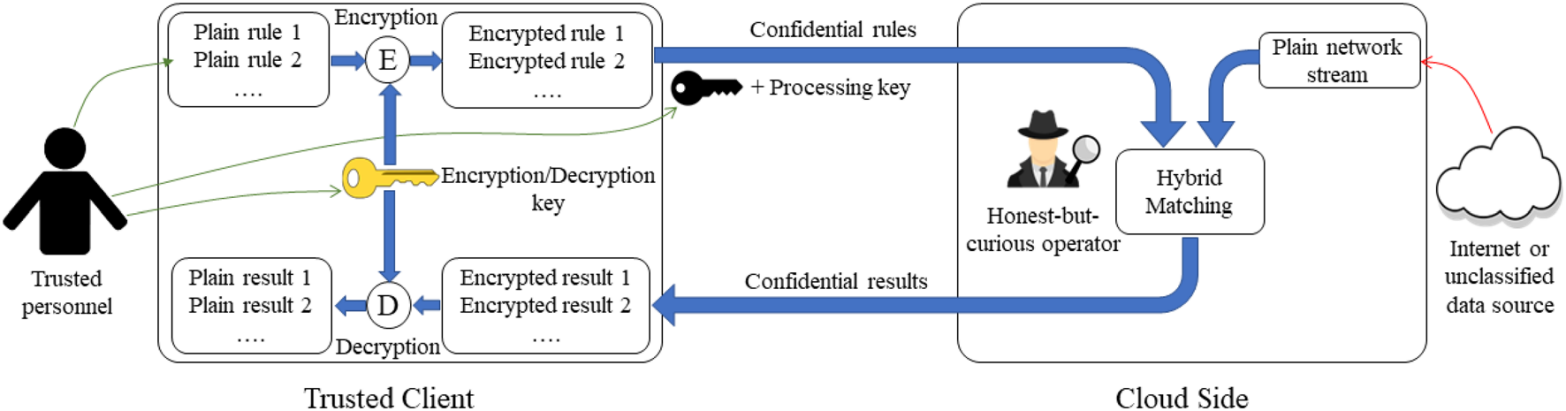
Oblivious NIDS system overview.

### Organization

The rest of this paper is organized as follows. Section “System model and requirements” shows the proposed system model along with the system requirements. Section “Related work” summarizes the related work. Section “HE hybrid plain-cipher binary gates” describes the idea and implementation of hybrid gates. Section “Hybrid rules matching algorithms” demonstrates the utilization of hybrid gates in implementing two sample oblivious NIDS fundamental machines; The Hybrid Direct Matching (HDM) and Hybrid Range Matching (HRM) machines with different versions. Section “Security analysis” discusses the adversary model and its goals along with the security propositions of the proposed system and their fulfillment proofs. Section “Performance acceleration with parallel processing” demonstrates a proposed parallel model implementation of HDM machine on GPU. The experimental setup is presented in section “Experimental setup and benchmarking results” along with performance measurements for the different versions of the hybrid machines implemented with different libraries on different platforms. The section also compares our approaches with the state of the art, illustrating the strength points of each and where each one is most suitable. A discussion of the proposed system capabilities and limitations is presented in section “Discussion”. Finally, the paper is concluded in section “Conclusion”.

## System model and requirements

In this section, we present an overview of the oblivious NIDS model with a hybrid matching layer and its essential requirements.

To achieve the idea of encrypted NIDS rules and results, we use the system overview depicted in Fig. 1. There are two involved parties; namely, the Trusted Client which represents the rules creator and the Cloud Side which represents the rules evaluator. The rules are created in plain at the Trusted Client and then encrypted using a private key. The encrypted rules are sent, over a public unsecured channel, to the Cloud Side for matching against the plaintext network traffic. The matching process results in an encrypted result, which is sent back to the Trusted Client, over a public unsecured channel, for decryption and taking necessary actions. Hence, the core research problem of this solution is to match plaintext network traffic against encrypted rules to generate encrypted matching results. For this model, the fundamental requirements are:

**MR1**: **Data Access** *(A) The rules and the matching results are comprehensible only to the Trusted Client and shall be encrypted and incomprehensible to the Cloud Side. (B) Trusted Client has no access to the plain network stream* There are two parties participating in the process; the Trusted Client and the Cloud Side. The Trusted Client creates and encrypts the rules and has no access to the plaintext data on the Cloud Side. On the other hand, the Cloud Side has access to the plain network stream and matches it against the encrypted rules producing encrypted results that can be decrypted only by the Trusted Client.
**MR2**: **No Intermediate Communications** *Once the encrypted rules are transmitted from the Trusted Client to the Cloud Side, no further communications shall be required except for sending back the results.* Any intermediate communications and bandwidth consumption would degrade the system's practicability or even render the system completely useless, as sending the plain network stream itself to the Trusted Client may require less traffic in this case.
**MR3**: **Small Result** *The size of the encrypted result sent back to the Trusted Client shall be smaller than the plain network stream size.* The encrypted matching result should be as small as possible, at least smaller than the plaintext size, for the same reasons as in MR2.
**MR4**: **Reusability** *An encrypted rule shall be reusable*. Reusing a rule against different plain network stream blocks must not leak any information about the rule, the matching results, or the relationship between the rules to the Cloud Side.
**MR5**: **Modularity** *The scheme shall provide a computationally efficient method to combine different independent and reusable sub-rules into complex rules.* Since each rule is typically composed of many small conditions, denoted here as sub-rules, e.g. source and destination IP and port numbers, protocol, and conditions on the payload, the scheme shall provide a computationally efficient method for the cloud side to reuse the matching result of each condition/sub-rule across many rules. I.e., matching against a specific source IP address shall happen once in the system, regardless of how many rules are using this condition. In other words, the matching machines shall be designed as independent and reusable modules. This provides the flexibility required by modern NIDSs to implement complex rules.

## Related work

One important field of research that is in close proximity to our research problem is Secure Multiparty Computations (MPC/SMPC)^9^. In SMPC environment, there are multiple parties who want to jointly calculate a shared function over their secret inputs without revealing their inputs to each other. After performing an SMPC protocol, only the result is revealed to all participating parties. However, none of the currently used SMPC protocols satisfy our model requirements stated in section “System model and requirements” as shown next.

Yao’s garbled circuit technique (GC)^10–12^ and its improvements^13–15^ are a generic SMPC protocol that is designed to evaluate any logic function between multiple parties. This protocol adopts an encrypted look-up tables approach to represent logic gates. One party, called the garbler, is responsible for creating and encrypting the circuit by creating suitable tags for all wires and providing encrypted look-up tables for the circuit gates according to these tags. The garbler then sends the encrypted look-up tables, without the wire tags, and its input tag to the other party; the evaluator. The evaluator receives the encrypted circuit and asks the garbler for their input tag using an Oblivious Transfer (OT)^16,17^ protocol so that the garbler does not know what the evaluator’s input is. Then, the evaluator evaluates the circuit gate by gate, without being able to comprehend any intermediate result, till they get the final result, which is shared with the garbler. In the problem of O-NIDS the look-up tables defy MR4 (Reusability) since the output space depends on the size of the look-up table of the output gate. An adversary may try different input combinations on the same circuit to construct a relationship between the inputs and outputs of the circuit, which compromises the rule secrecy. One other problem when trying to use this approach with our model is that it does not provide MR5 (Modularity). The evaluator has no way to combine the results of the sub-rules matching machines.

Goldreich, Micali, and Wigderson provided another famous generic SMPC protocol (GMW protocol)^18,19^ for evaluating logic functions securely between multiple parties. The core idea of GMW protocol is that each of the participating parties has an additive share of the value of every wire in the circuit under execution. At any time, when the parties decide to reveal the plain value of any wire, usually the output wire, they simply broadcast their secret shares of this wire and add them. In GMW protocol, evaluating a logic gate means calculating an additive secret share of the output wire for each of the participating parties. For XOR and NOT gates, the parties can evaluate the gates and calculate their output’s secret share locally without communicating with other parties. However, in the case of AND, OR, NAND, or NOR gates, the parties must communicate and apply Oblivious Transfer to evaluate the gate. In the problem of O-NIDS, these communications contradict MR2 (No Intermediate Communications), as the Trusted Client and the Cloud Side would have to communicate for all gates except XOR and NOT gates, which nullifies the usefulness of the system. A similar SMPC protocol for arithmetic circuits was developed by Ben-Or, Goldwasser, and Wigderson (BGW protocol)^20^. The key difference here is that the BGW protocol utilizes the concept of Shamir’s secret shares^21^ instead of additive shares. Similar to GMW, the secret shares of the addition gates outputs can be calculated locally by the participating parties. However, communication using Oblivious Transfer is required for evaluating multiplication gates which fails MR2. In addition, since both GMW and BGW use deterministic encryption schemes, reusing the circuit, i.e. rule, with different plain network stream inputs would allow the operator to gain information about the relationship between the circuit inputs and outputs, which essentially compromises the rule’s secrecy and fails MR4.

Kolesnikov^22,23^ presented a Gate Evaluation Secret Sharing (GESS) scheme which can be thought of as the information-theoretic analog of Yao’s garbled circuit. GESS allows two parties to secretly evaluate a Boolean Formula. For a boolean gate G, the output wire is assigned two secret values to represent the two possible outcomes. Each wire of the two input wires is then given two labels (total of 4 labels) such that the valid combination between any two labels from the two input wires can be used to reconstruct the label of the output wire. Thus, GESS exhibits similarities to Yao’s GC but distinguishes itself by eliminating the need for garbled tables. This characteristic positions GESS as a generalization of Yao’s GC. The problem with GESS is that its label size increases with circuit depth. Also, like Yao’s GC, it contradicts MR4 due to the usage of deterministic encryption schemes. It also doesn’t satisfy MR5 (Modularity), as there is no way to combine the results of the sub-rule matching machines. It should be noted here that both Yao’s GC and GESS require Oblivious Transfer at the input gate to allow the evaluator to get its input label secretly from the circuit creator. In our application, this Oblivious Transfer can be eliminated since the evaluator’s input, i.e. the plain network stream at the Cloud Side, is not a secret so no need for Oblivious Transfer, and the creator can send all possible input labels for the evaluator along with the circuit/rule.

The protocols of Private Set Intersection (PSI)^24,25^ represent important custom SMPC protocols that share a close idea to our model. PSI allows the participating parties to evaluate the intersection between their secret datasets without revealing anything else. In the O-NIDS problem, PSI does not satisfy MR1 (Result secrecy) because they were designed to reveal the intersection to all parties. PSI also contradicts MR3 (Small Result) and MR5 (Modularity), since it is conceptually designed to find and send an answer for all matched elements (intersection) so there is no clear way to aggregate the sub-rule matching results into single final result.

Another possible solution to the O-NIDS problem that could be considered is to use program obfuscation. Program obfuscation is an already-existing research problem that mainly targets protecting trade secrets and intellectual property and obstructing the attempts to reverse engineering privately owned software artifacts. Program obfuscation exists for different evasive functions and has numerous schemes, as in^26–28^ for obfuscating Boolean conjunctions,^29^ for obfuscating simple pattern matching, and^30^ for obfuscating Deterministic Finite Automatas (DFAs). The target of program obfuscation is to protect the evaluating circuit, but the default is to have the result readily available in plaintext at the evaluating system. This violates MR1 in the O-NIDS application, where the result needs to be kept encrypted and can only be evaluated by a remote trusted entity with access to the decryption key.

There are some preliminary attempts proposed in the literature for implementing a privacy-preserving NIDS but they did not reach practical performance metrics. Niksefat et al.^31^ used SMPC to securely evaluate an Oblivious Deterministic Finite Automata (ODFA), where a server (i.e. Trusted Client) creates the ODFA and a client (i.e. Cloud Side) evaluates it on plain network stream. However, this model protects only the OFDA but not the matching result which contradicts MR1. A survey of privacy issues in NIDS ^32^ studied over 27 IDS designs with some privacy properties and concluded that privacy-preserving signature-based NIDS is still an open problem that requires some advances in HE libraries. Sgaglione et al. ^33^ introduced a complete IDS capable of protecting the signature rules using homomorphic encryption, but they proposed to encrypt the plain network stream before comparison against the encrypted rules. The proposed solution required around 12 seconds to process a plain network stream of only 8 bytes.

Genise, Gentry, Halevi, Li, and Miccianciot [GGHLM]^34^ presented a promising modified somewhat homomorphic encryption scheme that enabled executing encrypted non-deterministic finite automata (NFA) over plaintext, which can be used into a privacy-preserving NIDS. Their application takes a regular expression (or regex) as input and then creates an NFA that implements this regex by assigning every letter in the regex alphabet an encrypted transition matrix with predefined dimensions according to the used parameters and definining the NFA’s state as an encrypted vector of a relevant length. The Cloud Side has to pick the corresponding encrypted transition matrix for each input letter in the plain network stream, do matrix $\times$ vector multiplication process, and send back the final state vector to the Trusted Side for verification. While this approach presents a high cloud searching speed, the length of the network traffic is limited and must be pre-defined before deployment. Also, the rule encryption time is very long preventing this solution from being used in adaptive NIDSs^35–37^. Additionally, the solution is not modular (MR5) and there is no way to combine results of sub-rules matching machines which is a typical feature of modern NIDSs. Other performance metrics, advantages, and drawbacks of this scheme are discussed in section “Experimental setup and benchmarking results”.

It should be noted that the secrecy principle of our proposed solution lies in hiding the data, i.e. the signature or the rule for which the NIDS is searching, not the evaluation circuit. This is different from the GGHLM approach that aims to hide the circuit and the data it processes.A summary of the compatibility of the major schemes from the literature with our model requirements is shown in Table 1.

**Table 1:** Compatibility of related schemes from the literature with the proposed model requirements.

Algorithm	Yao’s GC^10^	GMW ^18^	BGW^20^	GESS^22^	PSI^24^	Niksefat^31^	GGHLM^34^	Ours [this paper]
Approach	Logic functions	Logic functions	Arithmetic functions	Logic functions	Custom protocol	ODFA	NFA	Logic functions
MR1 data access	✓	✓	✓	✓	✕	✕	✓	✓
MR2 No Int. Comm.	✓	✕	✓	✓	✓	✓	✓	✓
MR3 small result	✓	✓	✓	✓	✕	✓	✓	✓
MR4 reusability	✕	✕	✕	✕	✓	✓	✓	✓
MR5 modularity	✕	✓	✕	✕	✕	✓	✕	✓

## HE hybrid plain-cipher binary gates

The term “hybrid-gate” is introduced here to refer to any binary gate that performs a logical operation between plaintext bit(s) and ciphertext bit(s) and produces an encrypted output. Hybrid gates are not supported in any HE library that targets binary encoding like TFHE ^5^ and FHEW ^38^ because, at first glance, the output will be Transparent. For example, if a plaintext input to an AND gate is 0 the output must be the encryption of 0 regardless of the value of the encrypted input. The ciphertext output would be transparent to the adversary if any non-linear gate (AND, NAND, OR, NOR) is used. Here we introduce a secure *multiplexer* implementation for both linear and non-linear hybrid gates as presented in the following subsection.

### Hybrid logic gates

**Figure 2:**
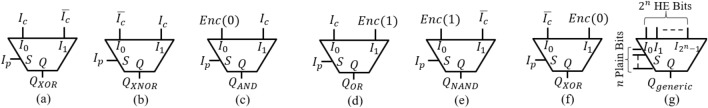
Multiplexer implementations for hybrid (**a**) XOR, (**b**) XNOR, (**c**) AND, (**d**) OR, (**e**) NAND, (**f**) NOR, and (**g**) generic logic gates.

XOR and XNOR gates are linear gates, i.e. if the plaintext input is fixed to 0 or 1, the output would be the same as the encrypted input or its complement, respectively. This can be used in constructing a multiplexer implementation of a hybrid XOR gate. The multiplexer is controlled by 1-bit of plaintext ($I_p$), while the multiplexer inputs are the 1 homomorphically-encrypted bit ($I_c$) and its complement ($\overline{I_c}$). The output of the gate, $Q_{XOR}$, represents the encrypted result. The input arrangement follows from the truth table, Table 2, and the proposed gate is illustrated in Fig. 2a and can be expressed as in (1). Since the value of the encrypted input is unknown with an equal probability of being 0 or 1, the value of the output will be equally unknown with the same probabilities. This means that the adversary cannot deduce any information about the output by controlling the value of one input and the output won’t be transparent. Hybrid XNOR gates can be implemented similarly as shown in Fig. 2b.

1 $$\begin{aligned} Q_{XOR}= {\left\{ \begin{array}{ll} I_c, &{} \text {if } I_p = 0 \\ \overline{I_c}, &{} \text {if } I_p = 1, \end{array}\right. } \end{aligned}$$

**Table 2:** XOR and XNOR truth table.

	$I_p$	$I_c$	$Q_{XOR}$		$Q_{XNOR}$	
$I_p=0$	0	0	0	= $I_c$	1	= $\overline{I_c}$
	0	1	1		0	
$I_p=1$	1	0	1	= $\overline{I_c}$	0	= $I_c$
	1	1	0		1	

The multiplexer implementation for the linear gates can also be extended to implement non-transparent non-linear gates as shown in Fig. 2. We assume that the Cloud Side is not provided with any known data, i.e., it cannot distinguish between the data pieces provided by the Trusted Client. Also, we assume that $\overline{I_c}$ is not computed by the cloud, so that $I_c$, $\overline{I_c}$, the encryption of 0 (*Enc*(0)), and the encryption of 1 (*Enc*(1)) are all oblivious inputs. The multiplexer inputs for the implementation of the hybrid gates are summarized in Table 3 which can be used to provide similar expressions like (1).

**Table 3:** Inputs of the multiplexer implementation for basic hybrid gates.

Mux input	$I_0$	$I_1$
XOR	$I_c$	$\overline{I_c}$
XNOR	$\overline{I_c}$	$I_c$
AND	*Enc*(0)	$I_c$
OR	$I_c$	*Enc*(1)
NAND	*Enc*(1)	$\overline{I_c}$
NOR	$\overline{I_c}$	*Enc*(0)

### Hybrid generic multi-inputs logic gates

The hybrid multiplexer concept can be extended to implement hybrid gates with more than 2 inputs, multi-input compound gates, and logic functions that have hybrid inputs. In general, a hybrid logic function with *n* plain input bits ($I_{p_i}$) and *m* HE ciphertext bits($I_{c_j}$) will be in the form of $f(I_{p_0},I_{p_1},\ldots ,I_{p_{n-1}},I_{c_0},I_{c_1},\ldots ,I_{c_{m-1}})$, with $2^n$ combinations for plaintext bits. For any plaintext bits combination *k*, the logic function can be reduced to a function $f_k(I_{c_0},I_{c_1},\ldots I_{c_{m-1}})$ with pure HE inputs only. This suggests the multiplexer implementation shown in Fig. 2g for the function *f*, where the multiplexer has *n* selectors, representing the plaintext inputs, and $2^n$ inputs where each input $I_k$ corresponds to a function $f_k$ of pure HE arguments. The functions $f_k$ can be realized with pure HE gates available in the HE library.

## Hybrid rules matching algorithms

In this section, we present two application algorithms that utilize the hybrid gates:

1. **Hybrid Direct Matching (HDM)**: Matching of plaintext stream against a homomorphically encrypted ciphertext rule. The algorithm describes how an oblivious NIDS at the Cloud Side can use the hybrid gates to search within the payload for an exact match against an encrypted signature rule.
2. **Hybrid Range Matching (HRM)**: Matching of a plaintext number against homomorphically encrypted range rule e.g. greater, less, within, and/or equal encrypted rule number(s). The algorithm shows how an oblivious NIDS at the Cloud Side can utilize the hybrid gates to implement range comparison rules, e.g. IP or port ranges, between an encrypted range rule and packet headers within the network stream.

Each of the two presented algorithms contains multiple variants that represent the flexible trade-off between speed and memory utilization. The resource requirements of these algorithms in terms of computation and communication complexity are also presented in this section and is quantified in section “Experimental setup and benchmarking results”. We also describe how to use these algorithms to implement a full practical rule and how to estimate its resource requirements. Finally, we present an approach to extend the application of the hybrid gates to generic hybrid logic circuits and hybrid Finite State Machines to implement regular expressions.

The proposed algorithms are comprised essentially of three parts:

- EncRules: This code runs on the Trusted-Client side to perform the following tasks:
  - Set the parameters of the underlying HE library by the trusted personnel.
  - Set the signature rules by the trusted personnel and encrypt them using the HE library to generate the encrypted rules.
  - Send the encrypted rules to the Oblivious-NIDS.
- Search/Compare: This code runs on the Cloud-Side to search through and/or compare the traffic against the encrypted rules to generate encrypted result bits. Here, all the hybrid gates of section “HE hybrid plain-cipher binary gates” can be used to perform direct matching, ranges, and wildcards as required by modern NIDSs.
- DecResult: This code runs on the Trusted-Client side to decrypt the result packet and generate an alert if necessary.

The first and last parts that include encrypting the rules and decrypting the result are quite similar for both algorithms; however, this is not the case with the second part as it depends on the required matching task. In all of the proposed algorithms, the O-NIDS does not need, nor it is able, to decrypt the rule, encrypt the plaintext stream, or understand the result.

For the algorithms demonstrated in this paper, it should be noted that SK and CK indicate Secret Key and Cloud Key, respectively. Also, a gate *x* denoted by $HHE _x$ means the hybrid gate *x*, which can be implemented in a way similar to (1) and Table 3. A gate *y* denoted by $HE _y$ is the homomorphic gate *y*, and its implementation depends on the homomorphic encryption library used. While the proposed implementations can be modified to accommodate when the plaintext word size is different from the encrypted rule word size, it is assumed here that they have the same size to reduce implementation-related complexity and give more focus on the concept.

### Hybrid direct matching (HDM) algorithms

The main aim of this algorithm is to search for an exact match of an encrypted word in the payload of the network stream. For this task, two variations that trade off speed for memory are presented.

#### Hybrid direct matching V1 (HDMv1)

In this variation, only the rule word is encrypted and sent to the cloud, which does all the required processing by utilizing the hybrid and fully homomorphic gates to match the encrypted word against all plain stream words, producing one encrypted sub-result bit for each plain word, and then aggregating the sub-result bits into a final result bit using fully homomorphic OR gates.

#### Hybrid direct matching V2 (HDMv2)

In this variation, the rule word itself is not encrypted; however, all the possible $2^{|word|}$ sub-result bits associated with matching the rule word against all possible $2^{|word|}$ plain words are encrypted and sent to the cloud. Hence, the Cloud Side does not have to calculate the sub-result bits for each plain word as it just needs to select them from the available sub-result bits array, and then aggregate the sub-result bits into a final result bit similar to HDMv1. Although this table lookup of the appropriate answer can work with any method that employs probabilistic encryption, only HE can support further processing on the sub-result bits to calculate a required final result and/or to aggregate them with results from other rules or algorithms. With this design, HDMv2 should have faster processing speed in the cloud at the cost of higher memory usage compared to HDMv1.

#### Implementation of HDM

Algorithm 1 describes the rule encryption at the Trusted Client side for the HDM. Depending on the HDM variation, either the rule word or the possible answers are encrypted. As an example use case, the rule can be assigned to be the 8-bit ASCII representation of the letter “A”. The HDMv1 will generate 8 HE-encrypted bits, each representing the encryption of 1 bit from the rule. However, HDMv2 will generate an array of $2^8$ HE-encrypted bits with the *Enc*(0) everywhere except at the index of the ASCII representation of “A” which gets the *Enc*(1). Algorithm 2 shows the hybrid searching process on the Cloud Side. In the case of HDMv1, the Cloud Side has to process each word from the plaintext stream against the encrypted rule word. As illustrated in algorithm 3, This is done by using the bit-by-bit hybrid XNOR between each bit in the plain stream word and the encrypted rule word and doing fully homomorphic AND between the XNOR result bits. On the other hand, HDMv2 skips the previous step as it has all the possible answers already pre-calculated so the Cloud Side has only to select the result bit for each plaintext stream word as demonstrated in step 6 of Algorithm 2. Following the previous use case, the Corpus is a sequence of plaintext network stream of 8-bit characters. $|\text {Corpus}|$ is the number of characters in the network stream that will be matched against the rule. The result of matching individual characters is reported in ResultCorpus, and the overall result bit is reported in EncResult. Regardless of the used variation, the overall result bit is calculated by aggregating the result bits of the hybrid words matching using fully homomorphic OR stage.

*HDMv2 as generalized hybrid gate*—It should be noted here that the proposed implementation of HDMv2 resembles a generalized hybrid gate as described in section “Hybrid generic multi-inputs logic gates” and represented in Fig. 2g. HDMv2 utilizes a 256 $\times$ 1 hybrid multiplexer with an 8-bit selector to implement the per-character matching circuit portion of HDMv1 (realized in software by the function IsEqualWord in Algorithm 3. The hybrid multiplexer inputs are the $2^8$ encrypted answer bits, while the 8-bit selector is the plaintext word that is being matched against the rule. This hybrid multiplexer with a fan-in of 256 is realized using a look-up table, where the table has 256 entries that resemble the multiplexer inputs, and an entry’s index represents the 8-bit value fed to the multiplexer selector lines. The circuit realization of IsEqualWord in HDMv1 consists of eight 2-bit hybrid XNOR gates + seven fully homomorphic AND gates, and the whole circuit is realized in HDMv2 by the 256$\times$1 hybrid multiplexer with 8-bit selector at the expense of greater rule size.

**Algorithm 1:**
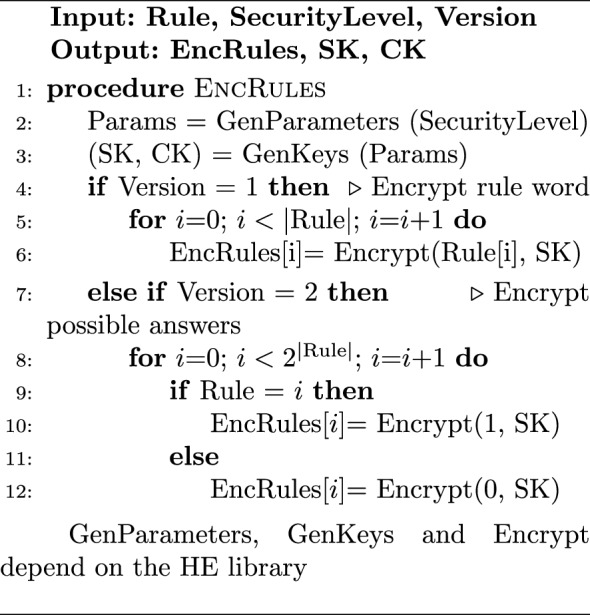
EncRules on Secure-Side-HDM

**Algorithm 2:**
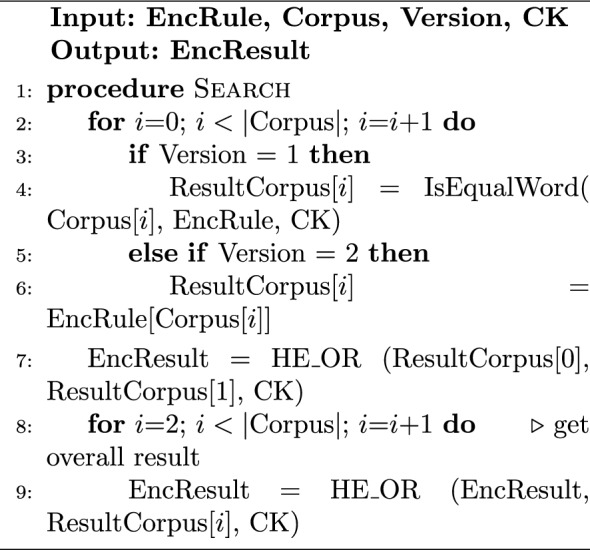
Search on Cloud-Side-HDM

**Algorithm 3:**
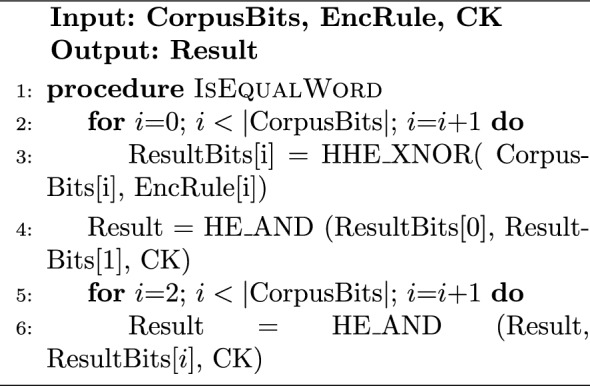
IsEqualWord on Cloud-Side-HDMv1

### Hybrid range matching (HRM) algorithms

The main aim of these algorithms is to perform Greater than (G), Less than (L), Range (R), and Equal (E) operations on a plaintext number against an encrypted rule described by a single confidential encrypted number in case of operations G, L, and E while using two confidential encrypted numbers in case of the R operation. In this context, the plaintext number can represent the port number in a packet header, while the encrypted number can represent the rule monitored by the NIDS. For this task, we present three possible variations that trade-off speed for memory.

#### Hybrid range matching V1 (HRMv1)

Similar to HDMv1, HRMv1 variation encrypts only the rule number in the case of G, L, or E operations or the two rule numbers in case of R operation at the trusted side and sends it to the Cloud Side which does all the required processing using combination of hybrid and fully homomorphic gates. The final result is produced as a single encrypted bit and sent back to the secure side for verification.

#### Hybrid range matching V2 (HRMv2)

HRMv2 variation follows a similar procedure as HDMv2, by pre-calculating and encrypting all the possible answers. For any specific rule number(s) and operation combination, there are $2^{|rule|}$ possible results to be calculated, encrypted, and sent to the Cloud Side. In this case, the Cloud Side will not have to do any processing as it just picks the encrypted answer from the possible results pool based on the plaintext number value under inspection and sends it back to the trusted side for verification or further aggregates it with other results if required.

#### Hybrid range matching V3 (HRMv3)

HRMv3 variation holds a middle ground between HRMv1 and HRMv2. If the rule number length is *n* bits, instead of encrypting all of the $2^{n}$ possibilities, HRMv3 divides the rule length into 2 parts of *n*/2 bits each: high part and low part. It then calculates and encrypts all possible answers for each part individually based on the rule’s value and required operation, stores them in two $2^{n/2}$ encrypted-bit arrays for E operation, or 3 arrays for G,L operations (additional auxiliary array is required in this case), and sends them to the cloud. The Cloud Side has to do a few processing steps to aggregate the selected sub-result bits from the $2^{n/2}$ encrypted-bit arrays into a final answer. Needless to say, HRMv3 can utilize any number of divisions $\ge$2; however, we selected 2 divisions as a sample of flexibility. Using this design methodology, HRMv1 should have the best memory requirements compared to the other variations, and HRMv2 should be the fastest while HRMv3 stands a middle ground between them in both memory and speed metrics.

#### Implementation of HRM

The rule encryption step for HRMv1 and HRMv2 is quite similar to HDMv1 and HDMv2 by encrypting the rule number/word itself or encrypting all possible answers, respectively, as previously shown in algorithm 1. The difference for HRMv2 is that the condition in step 8 of Algorithm 1 for a possible answer to be encrypted as 1 or 0 depends on the required operation being G, L, E, or R. HRMv3 has a slightly different rule encryption procedure, as the rule bits are divided into high and low parts with equal number of bits before encrypting the possible answers for each part individually. Algorithm 4 describes the rule encryption process for HRMv3 for the G operation. Following the same use case of section “Hybrid direct matching (HDM) algorithms”, we assume looking for an 8-bit input number from the network stream that is greater than 65 (0100 0001). Hence, the rule is “0100 0001”, RuleH will be “0100” and RuleL will be “0001”. EncRules will be a $3\times 2^4=3\times 16$ matrix. The rows of the EncRules matrix, EncRules[0][x], EncRules[1][x], and EncRules[2][x], represent the true condition for Index > RuleH, Index = RuleH, and Index > RuleL, respectively. Hybrid comparison at the cloud for HRMv1 approach utilizes gate-level minimized versions of the standard binary logic comparison circuits similar to the ones found in^39^. HRMv2 cloud task is similar to HDMv2 in algorithm 2 i.e. the cloud’s role is to pick the answer from the available answers pool. The hybrid comparison for HRMv3 with G operation is demonstrated in algorithm 5. Based on the plain number value, the cloud selects two partial results bit from the high and low possible answers pools along with one other bit from an auxiliary pool and combine these bits using HE gates into a final answer bit. Essentially, we are looking for a true condition at EncRule[0][PlainH] which represents PlainH > RuleH, or the conjunction (AND) of a true condition at EncRule[1][PlainH] which represents PlainH = RuleH and a true condition at EncRule[2][PlainL] which represents PlainL > RuleL.

The verification step is the same in all HDM and HRM algorithms; the encrypted result bit is sent back to the Trusted Client for decryption and verification, as described by Algorithm 6. It should be noted that in all HRM versions, the implementations for operations other than G follow similar procedures and can be deduced from the described HRM algorithms.

*HRMv2 and HRMv3 as generalized hybrid gates*—Similar to HDMv2, HRMv2 is actually a generalized hybrid gate that utilizes a $2^{16}\times 1$ hybrid MUX with 16-bit plaintext number as a selector. On the other hand, HRMv3 uses three $2^8\times 1$ hybrid MUXs to implement the hybrid range matching functions, with the plaintext 16-bit number divided into two 8-bit slices used as selectors to these multiplexers (as described in Algorithm 5). Both HRMv2 and HRMv3 simplify the circuit in HRMv1 into the bigger hybrid multiplexers but at the cost of increased memory requirements for the same rule. As with the case of HDMv2, the massive fan-in circuits of HRMv2 and HRMv3 are realized with look-up tables.

**Algorithm 4:**
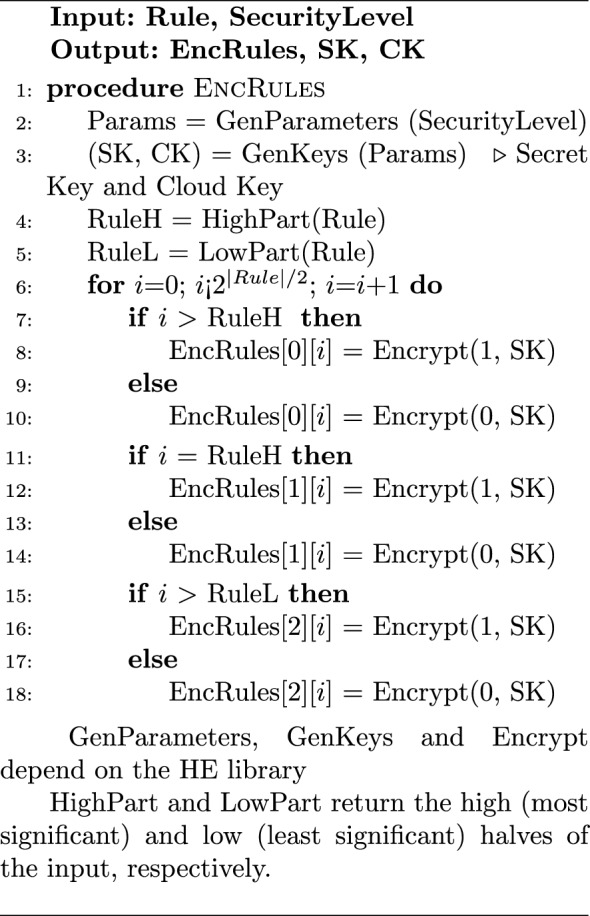
EncRules on Secure-Side-HRMv3-G

**Algorithm 5:**
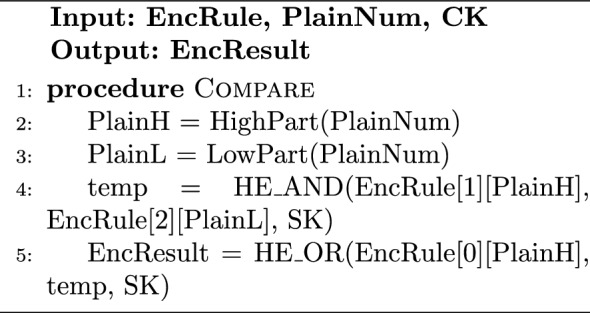
Compare on Cloud-Side-HRMv3-G

**Algorithm 6:**
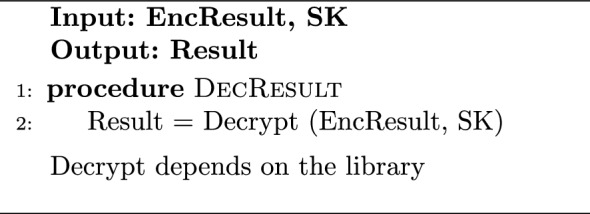
DecResult on Secure-Side

### Computational and communicational complexity of HDM, HRM algorithms

To calculate the computational and communicational complexity for HDM and HRM algorithms, we assume the number of bits per plain rule word to be *n* which is the same number of bits per plaintext network stream word. The number of plaintext network stream words is assumed to be *N*. For the computation complexity, we should note that the calculation time of the hybrid gates is negligible compared to the time consumed by the fully HE gates. This is because the execution time of the hybrid multiplexer that implements the hybrid gate is negligible (The output is a selection from the input) compared to the time-consuming bootstrapping operation that follows the fully HE gates (except for HE_NOT gate as it does not add noise and does not require bootstrapping) Thus, the calculation complexity is directly related to the HE gates while the other HHE gates and/or hybrid multiplexers are neglected in the calculations. On the other hand, the communicational complexity is proportional to the encrypted rule size, as the encrypted rule is transmitted from the trusted client to the cloud side to be used in the matching machine. The result bits that are sent back from the cloud side to the trusted client also contribute to the communication complexity; however, their effect depends on how frequently the trusted client needs the results to be communicated back. The computational and communicational analysis of GGHLM is also presented in section “Analysis of GGHLM”.

#### Analysis of HDM complexity

Execution of HDMv1 requires executing *n* HHE_XOR gates and $n-1$ HE_AND gates per word, and it requires $N-1$ HE_OR gates to calculate the final results for *N* words stream. On the other hand, HDMv2 skips the per-word HHE_XOR and HE_AND gates by pre-calculating their final results using 1 generic hybrid multiplexer per word (negligible execution time) and requires only $N-1$ HE_OR gates similar to HDMv1 to aggregate the per-word results into a final result. Thus, the speed gain of HDMv2 over HDMv1 can be described by (2) which indicates that HDMv2 is *n* times faster than HDMv1.

2 $$\begin{aligned} \lim _{N \rightarrow \infty } \frac{N(n-1) + (N-1)}{N-1} = n \end{aligned}$$

For the communicational requirements, HDMv1 requires *n* encrypted bits per rule word, while HDMv2 requires $2^n$ bits which is significantly larger then HDMv1. The computational requirements, i.e. # HE gates, and communication requirements, i.e. rule size as # of encrypted bits per rule, and the corresponding complexities of HDMv1 and HDMv2 are summarized in Table 4.

**Table 4:** Computation and Communication requirements and the corresponding complexities of HDM machines for n-bit rule and plaintext word size, matching with N plaintext words.

	Computation (Time)	Communication (Memory)
HDMv1	$Nn-1 \rightarrow {\mathcal {O}}(nN)$	$n \rightarrow {\mathcal {O}}(n)$
HDMv2	$N-1 \rightarrow {\mathcal {O}}(N)$	$2n \rightarrow {\mathcal {O}}(n)$

#### Analysis of HRM complexity

For Greater Than (G) or Less Than (L) operations, the execution of optimally minimized HRMv1 requires executing $(n-1)$ HE_AND gates, $(n-1)$ HE_OR gates, and *n* HHE_AND gates per plaintext number. HRMv2 executes only 1 big hybrid multiplexer per plaintext number. HRMv3 which divides the rule and plaintext word into two sections requires 3 big hybrid multiplexers, 1 HE_AND, and 1 HE_OR per plaintext number. For the Equal (E) operation, HRMv1 reduces to HDMv1 and requires the same number of gates. HRMv3 executes two hybrid multiplexers and 1 HE_AND per plaintext number, while HRMv2 remains the same as in the G or L operations. For the Range (R) operation, HRMv1 and HRMv3 require twice the number of gates if compared to G or L operations per plaintext number plus an additional HE_AND gate to combine the sub-results of G and L operations into R. However, HRMv2 still uses the same 1 hybrid multiplexer for the range operation.

All HRM machines, like HDM machines, require $(N-1)$ HE_OR gate to combine the per-number sub-results of *N* numbers into one final result. It should be noted that the execution of the hybrid multiplexers consumes negligible time.

Regarding the communicational complexity, HRMv1 requires *n* encrypted rule bits for G, L, and E operations, while it requires 2*n* encrypted rule bits for range operation. HRMv3 requires $3\times 2^{n/2}$ encrypted rule bits for G, L, and E operations, and requires twice this number for the R operation. Like HDMv2, HRMv2 requires $2^n$ encrypted rule bits for all operations. The computational requirements, i.e. # HE gates, and communication requirements, i.e. rule size as # of encrypted bits per rule, and the corresponding complexities of HRMv1, HRMv2, and HRMv3 are summarized in Table 5.

As an example to demonstrate the data in Table 5, HRMv1-R operation is achieved by combining the results of G operation on the lower range limit and L operation on the higher range limit using HE_AND gate. For matching a plaintext word with length *n* bits against a rule of the same length, the L operation requires $n-1$ HE_OR gates and the same number of HE_AND gates. G operation requires the same number of gates as L operation. An additional HE_AND gate is required to combine the results from both L and G operations. Thus, the total number of gates required for matching one plaintext word is $4(n-1)+1$. Combining the results of *N* words requires $N-1$ HE_OR gates and hence the total number of gates required for HRMv1-R operation on N words is $(4(n-1)+1)N + N-1)=2(2n-1)N-1 \rightarrow {\mathcal {O}}(nN)$. HRMv1-R requires the encryption of 2 *n*-bit rule words, this its memory requirements is $2n \rightarrow {\mathcal {O}}(n)$.

**Table 5:** Computation and Communication requirements and the corresponding complexity of HRM machines for n-bit rule and plaintext word size, matching with N plaintext words for Greater (G), Less (L), Equal (E), and Range (R) operations.

	#HE gates $\rightarrow$ computational complexity (Time)			# encrypted bits per rule $\rightarrow$ communicational complexity (Memory)		
	G,L	E	R	G,L	E	R
HRMv1	$(2n-1)N-1 \rightarrow {\mathcal {O}}(nN)$	$nN-1\rightarrow {\mathcal {O}}(nN)$	$2(2n-1)N-1\rightarrow {\mathcal {O}}(nN)$	$n\rightarrow {\mathcal {O}}(n)$		$2n\rightarrow {\mathcal {O}}(n)$
HRMv2	$N-1 \rightarrow {\mathcal {O}}(N)$			$2^n\rightarrow {\mathcal {O}}(2^n)$		
HRMv3	$3N-1 \rightarrow {\mathcal {O}}(N)$	$2N-1 \rightarrow {\mathcal {O}}(N)$	$6N-1 \rightarrow {\mathcal {O}}(N)$	$3\times 2^{n/2}\rightarrow {\mathcal {O}}(2^{n/2})$	$2\times 2^{n/2}\rightarrow {\mathcal {O}}(2^{n/2})$	$6\times 2^{n/2}\rightarrow {\mathcal {O}}(2^{n/2})$

#### Analysis of GGHLM

GGHLM processes the input plaintext *units* from the plain network stream by performing a Matrix $\times$ Vector operation for each unit. We use the term “unit” to describe the plaintext input chunk to the algorithm, as it may be the same or different from the term “word” we used for HDM and HRM earlier. Let *r* be the base of the number/character system used by GGHLM, this means it has *r* possible different symbols from the plaintext network stream. For example, for the binary base $r=2$ only 0 and 1 are expected as symbols while if the base is the English language alphabet capital letters then the expected symbols are A, B, C,..., and Z with $r=26$. This number corresponds directly to the number of transition matrices that should be encrypted at the trusted client and sent to the cloud side. Thus, the communication complexity of GGHLM becomes ${\mathcal {O}}(r)$ which is independent of the rule size.

The computation complexity of GGHLM is related to the number of Matrix $\times$ Vector operations. If we use the same terminology of *N* words and *n* bits per word as with HDM and HRM, then GGHLM would need to execute approximately $Nn/(log_2r)$ Matrix $\times$ Vector operations to process *N* words. This means the computation complexity of GGHLM is ${\mathcal {O}}(\frac{Nn}{log_2r})$. This indicates the trade-off in GGHLM, where increasing the base *r* increases the communicational complexity and reduces the computation complexity.

### Extension to complete rules and batching

As the proposed matching machines are built upon the elementary hybrid logic gates concept, this results in two important features:

- **Customizability**: Any of the HDM or HRM machines can be fine-tuned for a specific matching objective by changing the underlying HE logic circuit. In fact, a fully customized Finite State Machine (FSM) can be implemented using the hybrid gates to achieve a certain target.
- **Modularity**: The outputs of the matching machines can be easily combined using HE_AND or HE_OR as required.

The above-mentioned features allow the construction of complex rules easily by implementing a matching machine for each part of the complex rule and then combining the results using the appropriate HE gates.

For illustration, we consider a SURICATA rule^40^ that takes the form of (**alert protocol source_IP source_Port -> destination_IP/destination_subnet destination_Port **). In this case, the source IP can be an exact match or a range, which can be achieved by HDM and HRM machines respectively. It should be noted that the provided implementation of HDM machines in Algorithms 2 and 3 is designed to search for an encrypted rule word in plaintext corpus; however, it can be easily modified to direct match a certain number of rule words against the same number of plaintext words as may be required in an exact source IP matching and the same applies to the source port. On the destination side, the rule specifies a destination network in the above example. In this case, the Trusted Client may decide to take advantage of this to reduce the rule size, and hence the matching time, by matching only against the network address bits, or they can decide to provide more obfuscation by performing dummy matching against the host bits. Dummy matching can be achieved by implementing dummy hybrid gates that have all of their inputs as the encrypted value of the same plain value. The destination port can be treated as the source port, and the matching sub-results of each part of the rule can be then combined using HE_AND gates to get a final result. A numeric example to the requirements of a complete SURICATA rule is demonstrated in section “Extension to complete rule sets”.

One other advantage of the modularity feature is the ability to batch the results. A typical application of an oblivious NIDS may not require too frequent matching results for every network traffic element to be sent back to the Trusted Client. Rather, it may require the Cloud Side to keep watching for some certain rule in the incoming network traffic and send back a single result bit after a defined period of time to the Trusted Client to tell if there is a hit or not. If a hit was scored, the Trusted Client may decide to instruct the Cloud Side to drill down for the network traffic element that caused the hit by sending back the sub-result bits of a specific time period to the Trusted Client. This batching process can be achieved by collecting the matching result of the rule against the network traffic elements using HE_OR gates, and it is useful in lowering the required network traffic between the Trusted Client and the cloud server.

### Generalization to arbitrary circuits and regular expressions

Pattern-matching intrusion detection systems rely extensively on regular expressions, a fundamental concept in computer science and formal language theory. Regular expressions, as succinct representations of patterns in strings, can precisely define attack signatures and serve as the basis for identifying malicious activities within network traffic and system logs, making them essential for effective intrusion detection systems. Numerous attempts and approaches can be found in the literature to optimize the regular expression matching algorithms for intrusion detection, as surveyed in^41^. Implementing regular expressions in intrusion detection often involves utilizing finite state machines (FSM), also known as finite automata, which offer a systematic approach to processing and recognizing patterns. Finite state machines provide the necessary computational framework for interpreting regular expressions, enabling efficient pattern matching in large datasets^42^.

The multiplexer implementation of the hybrid gates provides a complete set of standard and generalized hybrid logic gates that take both plaintext and ciphertext inputs and produce ciphertext outputs. On the other hand, HE algorithms and libraries provide a complete set of standard fully HE gates that take ciphertext input and produce ciphertext output without decrypting the input or output at any stage. Combining hybrid gates, fully HE gates, and standard plain logic gates allows the construction of any hybrid logic circuit that can accept both plain and encrypted inputs and produce an encrypted output. Since an FSM is essentially a logic circuit and can be constructed using logic gates, a hybrid FSM can be built using a combination of hybrid and fully HE gates. Once the hybrid FSM is constructed, it can be used in hybrid-matching the regular expression against the plaintext data. The steps to implement a hybrid matching machine for a regular expression are as follows:

1. **Define the rule**: The trusted client defines the rule based on the intrusion detection requirements.
2. **Create the regular expression**: The trusted client converts the rule into a regular expression.
3. **Create the FSM**: The trusted client converts the regular expression into an FSM.
4. **Create the logic circuit**: The trusted client implements the FSM using hybrid and fully HE gates, defining the hybrid parts and fully HE parts.
5. **Create an encoding table**: The trusted client creates a look-up encoding table to encode plaintext input into ciphertext bits which will be used as input to the FSM created before.
6. **Send the FSM and the encoding table to the cloud side**.
7. **Encode the network stream**: The cloud side encodes the plaintext stream into ciphertext bits using the encoding table provided by the trusted client.
8. **Match the encoded stream**: The cloud side feeds the FSM with the encoded input, evaluates the circuit, and updates the FSM’s current state.
9. **Send back matching result**: The cloud side sends the encrypted FSM’s state back to the trusted client for decryption and decision-making as required.

**Figure 3:**
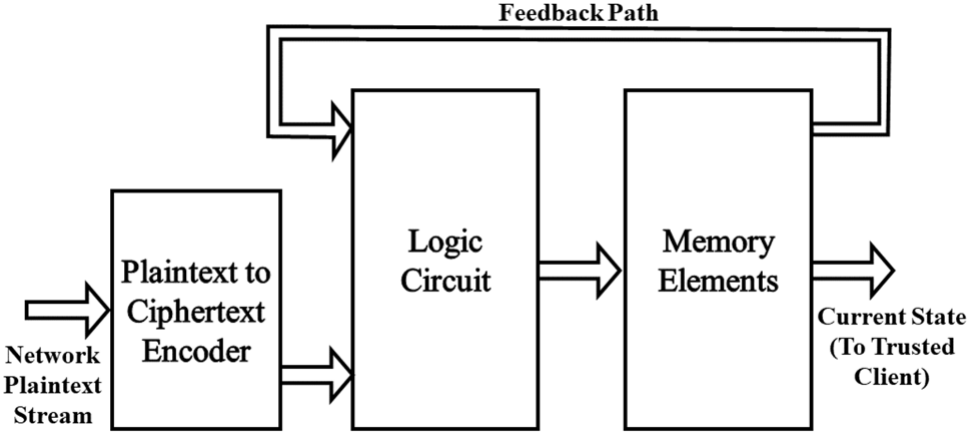
Block diagram of a generic hybrid logic circuit implementation of a regular expression as an FSM.

Figure 3 describes a generic block diagram for a hybrid logic circuit that implements a regular expression as an FSM. It should be noted here that the encoder results, the internal bits of the circuit, and the current status of the FSM are all ciphertext bits that cannot be decrypted or comprehended by the cloud side. However, the circuit elements and connections of the FSM can be reverse-engineered to reveal the structure of the regular expression even though the literals themselves are still encrypted. Thus, a dedicated security analysis and improvising technique to encrypt the regular expression structure are required to achieve the security goals of an ONIDS.

## Security analysis

In this section, we describe the adversary model and goals along with the expected attack surfaces. Then we discuss the security propositions against the adversary goals and demonstrate their applicability under the hybrid binary gates and the matching machines. Finally, we present a summary of the security of the adopted HE scheme (TFHE).

As mentioned in section “Introduction”, the two main entities in the system model are the Trusted Client and the Cloud Side. We focus mainly on the security at the Cloud Side.

### Adversary model

The potential adversary is the Cloud Side operator, which is modeled as an honest-but-curios operator^43^. The Cloud Side operator shall honestly follow all the instructions of the protocol, provide back the results, and has no intention of deceiving the Trusted Client or falsifying the results. In this context, the adversary’s main goals are:

**G1**: **Decrypt the encrypted secret rules**.
**G2**: **Decrypt the encrypted matching results**.

The main attack surfaces are:

**S1**: **The underlying HE scheme.**
**S2**: **Related encrypted rules segments.**
**S3**: **Related encrypted results.**
**S4**: **The matching circuit.**
**S5**: **Side-channel analysis of the matching process.**

The adversary is assumed to have access to all information being processed within the cloud server including physical side-channel leaks. For example, it may try to achieve the above-mentioned goals through techniques like:

- Intermediate Values Access: The adversary has access to all information being processed within the cloud along with step-by-step code execution values and results.
- Side Channel Analysis: The adversary is able to do any kind of passive side-channel analysis including power measurements, time measurements, and accessing memory locations.
- Code Reusing: While the honest adversary follows the code and provides the result back to the Trusted Client, it can rerun the code on its own with different inputs to try to deduct a relationship between the inputs and the outputs of a circuit/program.

### Security propositions

In order to defend against the adversary goals, the proposed system must preserve the secrecy of the rules and the matching results on the Cloud Side along with protection against any possible information leak that might happen during the process execution. In the following, we list the security requirements of the proposed system model with their fulfillment proofs.

**Adversary goal G1: decrypting secret rules**

#### Proposition 1

**(Rules Secrecy)** The encrypted rules processed with Hybrid Gates shown in Fig. 2 are as secure against decryption as the underlying HE scheme.

#### Proof

Every data bit in the rule is encrypted independently using HE at the Trusted Client side. From the perspective of the adversary, all rule bits have a 50% probability of being 0 or 1, and their encrypted values look different even if the plaintext bits share the same value. From the perspective of surface S1, the attacker has to directly break the underlying HE scheme to decrypt the rules. Also, the attacker gains no advantage through the circuit (S4) in this case, because processing the encrypted bits through Hybrid Gates does not change their probability of being 0 or 1. Hence, Hybrid Gates do not increase the advantage of an adversary in decrypting secret data bits in the rule and the encrypted rule is as secure against decryption as the HE scheme. The complexity of decrypting HE bits depends on the scheme and the chosen parameters. $\square$

The secret rule format depends on the matching machine version as shown in algorithms 1 and 4 where either the rule bytes are directly encrypted, as in the case of HDMv1 and HRMv1, or look-up tables that represents the rules, as the case in HDMv2, HRMV2, and HRMV3, are encrypted and sent to the Cloud Side. In either case, the Cloud Side receives the homomorphically encrypted rule bits as inputs to hybrid gates or as entries of look-up tables with equal probability of being 1 or 0 and with no visible similarity between any of them.

#### Proposition 2

**(Secure Rule Reusability)** Reusing an encrypted rule with related network traffic does not leak additional information to the Cloud Side operator.

#### Proof

The proposed implementation depends on the semantic security and probabilistic nature of the underlying HE scheme. Thus, even if the plain value $Decrypt(f(I_{p1},I_c))$ may be equal to $Decrypt(f(I_{p2},I_c))$, the operator can see only $f(I_{p1},I_c)$ and $f(I_{p2},I_c)$ which look completely different. This results from providing different encrypted values to all rule bits even if they have the same plain value as stated in the proof of Proposition 1. The only case where the results may look similar is when using the same plaintext against the same rule, which is a trivial case. $\square$

#### Proposition 3

**(Secure Usage of Related Rules)** Matching against related rules does not leak additional information to the Cloud Side operator.

#### Proof

As every rule bit is encrypted independently using an HE scheme, the Cloud Side operator would not be able to determine any similarity between the encrypted rules. Consequently, the Cloud Side operator would not be able to perform related rules attacks. $\square$

In the case of complex or complete rules as shown in section “Extension to complete rules and batching”, different rules may reuse similar sub-rules, e.g. same source IP address so the adversary may make use of the S2 surface. In this case, the adversary can understand that the same component is used in different rules, but it is still not able to decrypt the rule or that specific component. This is a direct trade-off for modularity of the proposed system and rule reusability. If absolute no leakage is required, the trusted client can encrypt every rule independently even if there are common components. This can be done by preventing re-using sub-rules in multiple rules i.e. each rule has its components/sub-rules encrypted independently even if this leads to encrypting the same plain sub-rule multiple times. In this case, the common components would look different in the homomorphic domain and the adversary would not be able to construct a relationship between these rules. However, this will come at the price of increased memory usage of encrypting the same component multiple times.

**Adversary goal G2**

#### Proposition 4

**(Secret Result)** The output of any Hybrid Gate is as secure against decryption as the underlying HE scheme.

#### Proof

The multiplexer implementation of the hybrid gates essentially selects one of the input bits to be the output bit. So, the output bit would have a 50% probability of being 1 or 0 because each of the input bits has the same probability of being 1 or 0 as shown in the proof of proposition 1. Hence, the adversary would be able to decrypt the result if and only if s/he is able to decrypt the underlying HE bits. $\square$

The proposed algorithms in section “Hybrid rules matching algorithms” have either a pure hybrid binary gate/function structure, as the case with the look-up approach in HDMv2 and HRMv2, or a mixed structure with hybrid gates and fully HE gates, like in HDMv1, HRMv1, and HRMv3. The secret result of a pure hybrid gate/function flows directly from the secrecy of its inputs, as the multiplexer implementation selects one of the input cipher bits that are already covered by homomorphic encryption to be the output. Thus, utilizing S4 provides no advantage against the secret result here. On the other hand, the secrecy of the result of a fully HE gate flows from the underlying HE scheme. A mixed structure has its input layer comprised of hybrid gates, which does not change the secrecy of the input bits, followed by a fully HE gates layer. S3 also does not give an advantage to the attacker, because the encrypted results would look different even if their plaintext values are the same, following the same argument in proposition 2. Thus, the only attack surface that may help the adversary is S1, which has the complexity and security of the underlying HE scheme.

#### Proposition 5

**(Side-Channel Leakage Resistance)** The secret rules and encrypted results are secure against side-channel attacks as the underlying HE scheme during the matching process.

#### Proof

Applying side-channel analysis against any of the matching machines might reveal only the cloud key, which is already public, not the private key as the latter does not exist at the Cloud Side. Thus, both the rules and the encrypted result are secure against side-channel analysis as the underlying HE scheme. $\square$

This proposition applies to both G1 and G2, where the adversary here tries to utilize S5 to gain an advantage in decrypting the secret rules or results using side-channel analysis. There are some recent successful attempts in the literature to recover the private key of popular HE schemes like TFHE at the Cloud Side using side-channel attacks. In^44^, the authors demonstrated the idea of introducing curated perturbations in the ciphertext bits that are returned to the Trusted Client. Depending on the perturbation value, the Trusted Client may or may not be able to decrypt the encrypted bit. The attack depends on utilizing the feedback sent from the Trusted Client to the Cloud Side in case of decryption failure to recover the error or noise value around ciphertext bits and, consequently, the secret key. However, this attack considers essentially a malicious Cloud Side operator and a Trusted Client that sends feedback in case of decryption failure, which is not the case in our system and adversary models.

### Security summary of TFHE

As shown in the previous sections, The security of the proposed hybrid matching machines depends on the security of the underlying hybrid matching gates which, in turn, depends on the security of the underlying Homomorphic Encryption Scheme. In our case, the security of the TFHE scheme flows from the hardness of the Learning With Errors (LWE) problem^45^. Security of LWE scheme is considered an independent and more general topic from the design of TFHE. Hence, estimating the security of TFHE, similar to any other LWE scheme, relies on the active cryptographic community at large. One of the most popular and regularly updated tools to estimate the security of LWE schemes is the Security Estimates for Lattice Problems project, denoted here as the LWE Security Estimator^46^, started by the research work in^47^. The tool combines algorithms from different resources to attack a given LWE scheme. The algorithms include:

1. bkw: A variant of the Blum-Kalai-Wasserman algorithm^48^ that uses coding techniques to reduce the memory requirement and improve the running time.
2. usvp: A primal attack that uses lattice reduction to find a short vector in the secret key lattice and recover the secret key.
3. bdd: A primal attack that uses lattice reduction and Babai’s nearest plane algorithm^49^ to find a close vector in the secret key lattice and recover the secret key
4. bdd hybrid: A hybrid attack that combines bdd with a meet-in-the-middle technique to reduce the dimension of the problem and increase the success probability.
5. bdd mitm hybrid: A hybrid attack that combines bdd with a meet-in-the-middle technique and a birthday paradox technique to reduce the dimension of the problem and increase the success probability.
6. dual: A dual attack that uses lattice reduction to find a short vector in the error distribution lattice and decrypt ciphertexts.
7. dual hybrid: A hybrid attack that combines dual with a meet-in-the-middle technique to reduce the dimension of the problem and increase the success probability.

For each of these algorithms, the tool reports various metrics including:

1. rop: The number of ring operations required to execute the attack algorithm, expressed in bits of security. This is the main measure of the hardness of breaking the scheme.
2. red: The number of ring operations required to perform lattice reduction, expressed in bits of security.
3. svp: The number of ring operations required to solve the Shortest Vector Problem, expressed in bits of security.
4. mem: The memory requirement of the algorithm, expressed in bits.

The tool can also be used to scan a range of parameters using the class ParameterSweep. LWE Security Estimator is still evolving and new algorithms and/or metrics are being added as they become available. In the context of TFHE, we work primarily with different types of ciphertexts: Learning With Error on the Torus (TLWE) and Ring Learning With Error on the Torus (TRLWE) (RLWE sample has a similar construction to the LWE sample but it is defined over polynomials instead of real numbers). Both are required to perform bootstrapping. In order to estimate the security of these parameters, we evaluate the LWE Security Estimator on the TLWE parameters as well as the TRLWE parameters and opt for the smallest rop of the two schemes from all the studied attack algorithms.

The parameters of TLWE and TRLWE in the default configuration of^50^, which we used, are defined in the following list and their values are presented in Table 6 along with the security estimates evaluated by^46^.

- *q*: Modulus of the space ${\mathbb {Z}}/q{\mathbb {Z}}$ of integers for the LWE samples. It represents how many bits are used to represent each coefficient in the TLWE and TRLWE samples. The higher *q*, the higher the security and computation time.
- *n*: Dimension of the LWE sample vector. *n* represents the length of the secret key in bits. The higher *n*, the higher the security and computation time.
- $e_{tlwe}$: Standard deviation of the Gaussian distribution function for the TLWE error term. A very low value of $e_{tlwe}$ is not secure and a very high value will force a decryption failure. LWE Security Estimator must be used to find a good value.
- *k*: The number of polynomials in the TRLWE sample.
- *N*: The order of each polynomial. *N* Efficient bootstrapping relies on *N* being a power of 2. $N \times k$ represents the length of TRGSW key that is used to encrypt the secret key for bootstrapping.
- $e_{trlwe}$: Standard deviation of the Gaussian distribution function for the TRLWE encryption.
- pbs_base_log: Specifies the base of the decomposition for the Polynomial Bootstrapping (PBS) operation, i.e., the Blind Rotation.
- pbs_level: Specifies the number of levels in the decomposition for the Polynomial Bootstrapping (PBS) operation.
- ks_base_log: Specifies the base of the decomposition for the Key-Switching (KS) operation.
- ks_level: Specifies the number of levels in the decomposition for the Key-Switching (KS) operation.

**Table 6:** Default TLWE and TRLWE parameters of TFHE^50^.

Parameter	TFHE
*q*	$2^{32}$
*n*	630
$e_{tlwe}$	$3.051\times 10^{-5}$
*k*	1
*N*	1024
$e_{trlwe}$	$2.98\times 10^{-8}$
pbs_base_log	7
pbs_level	3
ks_base_log	2
ks_level	8
TWLE-rop	$\sim 2^{124.5}$
TWLE-red	$\sim 2^{124.5}$
TRWLE-rop	$\sim 2^{126.6}$
TRWLE-rop	$\sim 2^{126.6}$

The security estimator results presented in Table 6 show a minimum security estimate of 124 bits for TFHE, which comes from the number of ring operations required by the attack on TWLE and for the lattice reduction.

## Performance acceleration with parallel processing

The abstracted dependence of the hybrid gates approach on the underlying HE scheme allowed us to utilize and test GPU-accelerated HE libraries to enhance the performance of our algorithms. In this section, we present the implementation details of HDMv1 on top of the GPU accelerated library cuFHE as a sample demonstration of the implementation flexibility of the hybrid gates approach and to show the achievable performance gain.

cuFHE^8^ is a bootstrappable FHE library that implements the TFHE scheme on Compute Unified Device Architecture - CUDA enabled GPUs utilizing the improved CUDA implementation of the numbertheoretic transform (NTT) in cuHE library^51^. cuFHE is a binary-based FHE library with an API that provides simple & high-level basic functions for basic HE operations. Basic operations like key generation, encryption, and decryption are performed on CPU, while CUDA implementations are provided for the actual Boolean operations functions like AND, OR, ... etc to allow executing these operations on GPU. Compared to TFHE, cuFHE does not provide a time-accelerated implementation for any single gate on its own; however, it allows the execution of multiple gates in a parallel fashion on the GPU which reduces the average required time by each gate as a result. This means the speedup gain from cuFHE depends on the HE circuit design itself; a circuit that can be executed in parallel paths would achieve a speedup gain while a circuit that adopts serial flow of the data bits will not benefit much if implemented using cuFHE.

**Figure 4:**
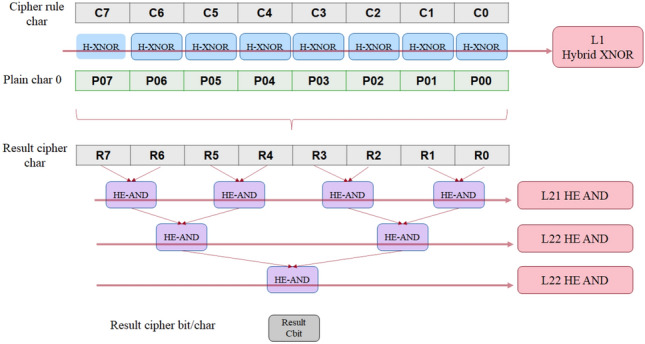
A section of the parallel model of HDMv1 for a single word hybrid matching.

The parallelization model depends on executing the maximum possible number of independent Boolean operations (gates) in parallel. In such implementations, the circuit must be viewed from a gate-level perspective. The circuit is divided into levels, where each level contains a number of independent gates that should be executed in parallel. The execution of each level should be finished before descending to a lower level to achieve data availability and synchronization between the levels. An optimum design should contain the smallest number of levels to limit the sequential execution and waiting time between levels. On the other hand, each HE gate execution in cuFHE is assigned an independent kernel launch, and the maximum possible number of parallel kernel launches depends essentially on the available hardware, i.e. the number of Streaming Multiprocessors (SMs), which directly limits the maximum number of gates to be executed in parallel. Therefore, the designer must be careful in tuning the number of levels and number of gates per level to achieve the best performance. Figure 4 illustrates a section of the adopted parallel model of HDMv1 for matching a single 8-bit plain word against an encrypted rule word with the same size. The first level, L1, is the hybrid XNOR (HHE_XOR) between the plain character bits and the cipher rule bits which utilizes the hybrid MUX model described in section “HE hybrid plain-cipher binary gates”. These HHE_XNOR operations can be executed in parallel in a single level as they are independent. The next step is to perform HE_AND operations between all the result bits from the previous level, if all the bits are 1s then there is a match, if any bit is 0 then this is a miss. As can be seen in Fig. 4, the HE_AND operations are executed in tree fashion to maximize the number of operations per level and minimize the number of levels. The final result is a cipher bit that indicates whether there is a match or not.

**Figure 5:**
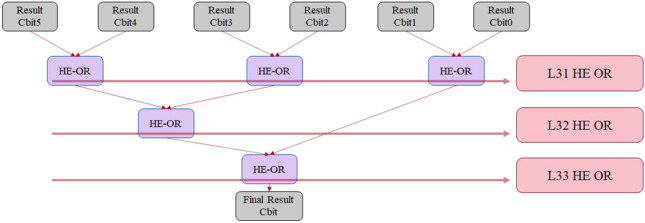
Parallel aggregation model of HDMv1 for the result bits of hybrid matching of 6 plain words.

This matching process can be executed for multiple plain words (string) at the same time to maximize parallelization and efficiency. As the matching process produces a cipher bit result for each word, an HE_OR operation can be executed on all result bits to get a single final result bit that indicates if there is a match in the string under processing. To demonstrate the process, Fig. 5 shows the circuit section that implements the HE_OR operation between 6 result bits, as an example, in a tree fashion similar to the previous HE_AND stage to maximize parallelization and efficiency. The circuit can be extended to accommodate for any number of bits. The required number of gates in each level of the matching process of N 8-bit plain words against one encrypted rule word with the same size is presented in Table 7 as a reference.

**Table 7:** Required number of gates per each level in the hybrid matching process of N plain 8-bit words with a single encrypted word rule with the same size.

Level	Gate type	Number of gates
L1	H-XNOR	N*8
L21	HE-AND	N*4
L22		N*2
L23		N
L31	HE-OR	$\lceil$N/2 - 0.5$\rceil$
L32		$\lceil$N/4 - 0.5$\rceil$
...		...
L3x		$\lceil$N/$2^x$ - 0.5$\rceil$

## Experimental setup and benchmarking results

In this section, the experimental setup, configuration settings, and the parameters used to test the algorithms in section “Hybrid rules matching algorithms” and the parallel model in section “Performance acceleration with parallel processing” are presented. Benchmarking results for the proposed algorithms variations, GGHLM approach variations, and the parallel model are also demonstrated. The benchmarking methodology targeted four objectives:

1. Implementing one of the proposed algorithms using different HE libraries on CPU to compare their performance and to demonstrate the library-independency of the hybrid gates approach.
2. Performing a detailed benchmarking comparison on CPU between our approaches built on top of TFHE library and the current state-of-art, illustrating their pros and cons and highlighting the suitable operation applications for each.
3. Testing the applicability and resultant gain of implementing a sample of our algorithms in a parallel form on top of a GPU-accelerated HE library.
4. Demonstrating the practical resources requirements for matching against a complex rule as pointed in section “Extension to complete rules and batching”.

The code snippets were written using C++ since it is common between all the tested HE libraries. Also, the experiments were performed on a PC with a Core I9-10900 processor and 32 GB of memory with an Nvidia RTX 3090. Elementary calculations and measurements were performed on TFHE and GGHLM to get the time and memory requirements for the fundamental operations in both libraries. The results allowed theoretical extrapolation to the time and memory requirements of more complex operations at any scale. To confirm the extrapolated expectations, all HDM, HRM, and GGHLM implementations were executed on a single thread, and the maximum resident memory and total execution time were measured using “/usr/bin/time -v” Linux command.

### Performance evaluation under different HE libraries

To achieve the first target, HDMv1 variation is implemented using 3 different libraries: TFHE^50^, Microsoft SEAL (BFV scheme)^6^, and PALISADE (BFV, BGV, and TFHE schemes)^7^. It should be noted that BFV and BGV are integer-based schemes and were configured with modulo-2 arithmetic to fit with our implementations. Also, they are leveled schemes which means they support operations on ciphertext to a limited depth, that is configured by their parameters, compared to the bootstrabable schemes like TFHE that supports operations or circuits on ciphertext to any arbitrary depth. This is why the SEAL implementation of BGV scheme is not reported in this paper as it did not provide a sufficient multiplication depth suitable for our application within a reasonable choice of parameters. While CKKS scheme^52^ is implemented by SEAL and PALISADE, it is used for approximate computations on integers and complex numbers, which does not fit with our target application. FHEW scheme^53^ is also implemented in PALISADE; however, it is not tested because TFHE is considered an improved and faster version of FHEW ^50^. The HE libraries parameters in all of these implementations are chosen to ensure a minimum of 128 bits of security.

As mentioned in section “Hybrid rules matching algorithms”, the plaintext word size and the ciphertext rule size are chosen to be the same to not overcomplicate the implementation. The word size here is selected to be 8 bits, the rule is one encrypted word, and the plaintext stream consists of 10 words so the task would simulate the searching for 1 HE encrypted ASCII character in a stream of 10 plain characters. The results of this experiment are presented in Table 8 which shows a wide variety of search speeds. Even for the same HE scheme, e.g. BFV or TFHE, the searching speed is different based on the used library. It should be noted that for integer-based schemes, the searching algorithm requires a multiplication depth of at least 7 otherwise the decryption will fail. PALISADE-BFV with a depth of 7 shows the minimum searching time while PALISADE-BGV with a depth of 50 is the slowest. The multiplication depth has a significant impact on the performance. Increasing the multiplication depth (i.e. searching circuit depth) increases the searching time and the size of the encrypted bit. PALISADE-BFV scheme shows better performance than SEAL-BFV especially at high depths. It should be noted that TFHE library has infinite multiplication depth since it is a bootstrapable scheme, and it will eventually achieve better results for deeper circuits (e.g. longer plaintext searching strings) in addition to the independent encrypted bit size.

**Table 8:** Benchmarking results for implementing the hybrid search algorithm for 1-byte HE encrypted rule over 10-byte plaintext using TFHE, SEAL, and PALISADE libraries.

Library	Scheme	Base	Parameters	Multiplication depth	Search speed (ms)	Encrypted bit size (Bytes)
TFHE	TFHE	Binary Logic	$\lambda$ = 128	Infinity	1,051	**2536**
SEAL	BFV	Int. Arith.	Plaintext Modulus = 4096	4	Decryption Failed	88,488
			Plaintext Modulus = 16384	11	1061	432,382
			Plaintext Modulus = 16384	25	5187	1,826,173
			Plaintext Modulus = 32768	50	25,826	7,404,598
PALISADE	BFV	Int. Arith.	Mult. Depth = 4		Decryption Failed	396,249
			Mult. Depth = 7		**390**	396,249
			Mult. Depth = 11		1,240	1,313,983
			Mult. Depth = 25		4,499	4,722,315
			Mult. Depth = 50		19,743	17,830,435
	BGV	Int. Arith.	Mult. Depth = 4		Decryption Failed	658,638
			Mult. Depth = 7		1,378	2,100,775
			Mult. Depth = 11		2,254	3,149,811
			Mult. Depth = 25		16,434	13,637,181
			Mult. Depth = 50		120,160	53,485,944
	TFHE	Bin. Logic	$\lambda$ = 128	Infinity	11,089	4,141

Fastest search speed and smallest encrypted bit size are in bold.

### Performance comparison with state-of-art

To achieve the second objective, we compared the performance of our proposed algorithm bundles against the encrypted NFA approach of GGHLM targeting the two tasks presented earlier in section “Hybrid rules matching algorithms”, namely the Hybrid Direct Matching and the Hybrid Range Matching. For this purpose, all HDM and HDR variations are implemented using TFHE with configuration parameters that ensure a minimum of 128 bits of security. The elementary measurements for GGHLM showed that the encryption time for a transition matrix of a member in the regex alphabet is ~5 s, and its size is ~33 MB. A homomorphic matrix $\times$ vector operation takes ~5 ms. For TFHE on the other hand for our approaches, The encryption time of 1 bit is ~21 usec and its size is ~2.4 KB. A homomorphic binary gate execution takes ~13 ms. These numbers can be used to predict the time and memory costs of more complex operations of any scale, and they proved good accuracy compared to the actual measurements presented next.

#### Performance of the hybrid direct matching algorithm

For the HDM machine, the task is to search for an encrypted character rule in a stream of 10 plaintext characters, similar to the task of the first objective, so the rule/word size is selected as 8 bits. To perform the same task with GGHLM, a regular expression in the form of “.*c.*” is inputted to their implementation, where “.” represents a wildcard (The currently available implementation of GGHLM does not support the wild card “.”. So the wildcard was replaced by a logical OR between all 256 possibilities of an 8-bit word), “*” is the Kleene star, and “c” is the rule character. A random 10-byte string is fed to the NFA as input to represent the plaintext stream. The configuration parameters were chosen as suggested in GGHLM to achieve a minimum of 51 bits of security. While it is possible, theoretically, to change the parameters to achieve 103 security bits, the provided implementation^54^ did not allow that practically.

Table 9 shows the benchmarking result of the HDM task. While the key creation time is comparable between the HDM variations and GGHLM, the rule encryption time for GGHLM is in the order of minutes which is $10^6$ times greater than HDMv1 and $10^5$ times more than HDMv2. The latency in rule encryption makes GGHLM unsuitable with the adaptive NIDS environments that require frequent updates. For the searching speed on the cloud, GGHLM is 2 times faster than HDMv2; however, its maximum resident memory is >14 GB, which is more than 50 times the memory requirements of HDMv1 or HDMv2. Also, the encrypted rule size to be transmitted from the Trusted Client to the Cloud Side for GGHLM is more than 8 GBs, which is $10^5$ times greater than HDMv1 and $10^4$ times greater than HDMv2. This massive maximum resident memory makes GGHLM unsuitable for a practical NIDS that has thousands of rules running concurrently, and the huge rule encrypted size makes transmitting new rules frequently a cumbersome task for adaptive NIDSs. When it comes to the decryption speed of the encrypted result back as the Trusted Client, HDM variations are $10^5$ times faster compared to GGHLM. It is also important to notice that these measurements are taken while the configuration parameters allowed only 51 bits of security for GGHLM, changing the parameters to give higher security bits increases the resources consumption of GGHLM dramatically. It should be noted that HDMv2 is faster than HDMv1, but it comes with a larger encrypted rule size as expected. While the difference in the rule size has an effect on the transmission costs, it adds negligible memory load on the Cloud Side processing unit as the significant amount of the application’s memory is occupied by the cloud key and the algorithm itself.

**Table 9:** Performance comparison between HDMv1, HDMv2, and GGHLM for the task of direct matching a stream of 10 plain bytes with 1 encrypted rule byte.

Location	Operation	HDMv1	HDMv2	GGHLM^54^
Trusted Client	Key Create (Time)	856 ms		721 ms
	Rule Enc. (Time)	168 us	5370 us	22 mins
Cloud Side	Rule Match (Time)	1051 ms	120 ms	52 ms
Trusted Client	Dec. (Time)	1 usec		438 ms
Encrypted Rule (Size)		19.81 KB	634 KB	8.26 GB
Encrypted Result (Size)		2.48 KB		33. KB
Key (Estimated Size)		113.7 MB		59.1 MB
Max Res. Memory (Size)		253.1 MB	253.7 MB	14.15 GB
Security bits		128		51

#### Performance of the hybrid range matching algorithm

For the HDR machine, the task is to compare a plaintext number against an encrypted range rule. The encrypted range rule included the 4 common operations: Greater (G), Less (L), Equal (E), and Range (R). The plaintext and the encrypted rule word size is 16 bits, to be similar to a realistic IPV4 port size. To perform the same task using GGHLM, a specific regular expression has to be created to describe each rule number and operation combination. It should be noted that for GGHLM, the task can be addressed using different plaintext and rule number representation under different numbering systems/bases allowing for speed $\times$ time trade-off. For instance, the NFA can adopt the decimal representation of numbers so it would have 10 different symbols i.e. 10 different transition matrices. In this case, the plaintext input will be handled as a stream of 5 decimal digits to accommodate for the whole range from 0 to 65,535. One other way is to adopt the binary representation so the NFA has only two different symbols as inputs. In this case, the plaintext input number is interpreted as 16 successive binary bits/symbols. To achieve a thorough comparison with our approach and deeper insight into the performance of GGHLM, we implemented task using GGHLM with the two bases: decimal and binary.

Table 10 shows the benchmarking result of the HRM task implemented using HRMv1, HRMv2, HRMv3, decimal interpretation for GGHLM (GGHLM-D), and binary interpretation of GGHLM (GGHLM-B). The table also includes mathematically calculated metrics of a hypothetical GGHLM implementation with $2^{16}$ symbols (GGHLM-H16), for which the plaintext input number is treated as a single symbol. This is to mimic HRMv2 which has all $2^{16}$ answers pre-calculated. The results show that the key generation time is comparable for all approaches with the used libraries configuration, noting that changing GGHLM to get higher security bits increases the key generation time by a factor of 10. The rule encryption task is much faster in all HRM variations with 3 orders of magnitude for HRMv1 and HRMv3 and 1 order of magnitude for HRMv2 as compared to GGHLM. HRMv1 has the fastest rule encryption as expected for an encryption time of < 2 ms. GGHLM-H16 has unpredictable rule encryption time that extends to almost 4 days. For the rule matching at the cloud, HRMv2 has no competitor with an extremely fast 1.25 usec matching time and GGHLM-H16 comes in second place with much slower time of 5 ms. HRMv3 and GGHLM-D show comparable performance with HRMv2 being faster in E operation, same in G and L operations, and slower in the R operation, but it is faster than GGHLM-B in all operations. HRMv1 is the slowest among all candidates in the matching operations as it requires the maximum number of HE gates executions on the cloud. For the decryption time, all HRM variations have 1 usec decryption time which is $10^5$ times faster than GGHLM. For the data transmission between the Trusted Client and the Cloud Side, all HRM variations have smaller encrypted rule sizes compared to GGHLM. HRMv1 has the smallest rule size in the range of a few hundred KBs. HRMv3 comes in second place with rule size in the order of a few MBs. GGHLM-D and GGHLM2-B show encrypted rule sizes in the order of 330 and 66 MBs, respectively, while HRMv2 comes in a middle ground with 159 MBs. GGHLM-H16 gives an unpractical rule size in the order of TBs. The encrypted result sent back from the Cloud Side to the Trusted Client has a unified size for all HRM variations (an encrypted bit) of 2.48 KBs, which is relatively smaller than the size of the encrypted GGHLM result (encrypted state) of 33 KBs. All candidates show comparable Maximum Resident Memory size in the order of a few hundred MBs, except for GGHLM-H16 which comes with unpractical memory utilization in the TBs range.

**Table 10:** Performance comparison between HRMv1, HRMv2, HRMv3, GGHLM-D, GGHLM-B, and GGHLM-H16 for the task of matching a 16-bit plaintext number against encrypted range rules.

Location	Operation	HRMv1			HRMv2			HRMv3			GGHLM-D			GGHLM-B			GGHLM-H16*		
		G,L	E	R	G,L,E,R			G,L	E	R	G,L,E,R								
Trusted Client	Key Creation (Time)	826.3 ms									721.089 ms								
	Rule Encryption (Time)	1.311 ms	1.323 ms	1.974 ms	1342 ms			15.8 ms	10.4 ms	31.8 ms	50.79 sec			10.51 sec			3.926 days		
Cloud Side	Rule Matching (Time)	386 ms	192 ms	790 ms	1.25 us			25.7 ms	12.9 ms	64.9 msec	25.3 msec			79.49 msec			5.01 msec		
Trusted Client	Decryption (Time)	1 usec									497.703 msec								
Encrypted Rule (Size)		158.5 KB	158.5 KB	237.75 KB	158.5 MB			1.86 MB	1.24 MB	3.71 MB	330.3 MB			66.1 MB			2.064 TB		
Encrypted Result (Size)		2.48 KB									33.03 KB								
Estimated Key (Size)		113.7 MB									59.1 MB								
Max Resident Memory (Size)		253.31 MB	253.32 MB	253.61 MB	412.4 MB			255.1 MB	254.4 MB	257 MB	623 MB			172 MB			3.524 TB		
Security bits		128									51								

*Values for GGHLM-H16 are estimated using the elementary measurements for GGHLM as follows:

Rule encryption time = Time for encrypting one transition matrix $\times$ |Alphabet| = 5.08 s $\times$ $2^{16}$

Rule Matching Time = (Matrix $\times$ Vector) process time $\times$ #input symbols = 5.01 msec $\times$ 1. (There is a matrix for every possible plain number)

Encrypted Rule Size = |Alphabet| $\times$ size of encrypted matrix = $2^{16}\times$ 33.03 MB.

Maximum Resident Memory = 1.707 $\times$ Encrypted Rule size + Key Size = 1.707 $\times$ 2.064 TB + 59.1 MB.

The key creation time, key size, decryption time, and the encrypted result size are the same

### Performance evaluation of the parallel models

To achieve the third objective, the parallel model of HDMv1, presented in section “Performance acceleration with parallel processing”, is implemented with cuFHE library and tested on the RTX3090 hardware that has 82 Streaming Multiprocessors. For consistency with other measurements in this paper, the size of the plain word and rule word is chosen to be 8 bits as a character. The elementary measurements showed that the execution time of an HE binary gate is ~11 ms, and this result is used as a base to calculate the time requirements of more complex circuits, like the proposed parallel model of HDMv1, and proved a good accuracy compared to the actual measurements. The calculated number of gates and levels in Table 7 was used to theoretically predict the required matching time between 5, 10, and 50 plan 8-bit words against a single word rule of the same size given the measured single gate execution time on CPU and GPU. The execution time of the hybrid XNOR is negligible compared to the HE AND/OR operations, because it is a direct MUX operation with inputs pre-calculated, and it is ignored in the calculations. These estimations and practical measurements are presented in Table 11. It should be noted that the number of actual execution levels may be more than 1 per theoretical level because of the limited number of SMs in a GPU which limits the maximum number of concurrent gates. For example, the L21 AND level which contains 200 AND operations is executed in 3 actual levels because the maximum number of concurrent kernel launches for RTX 3090 is equal to the number of hardware SMs which is 82.

**Figure 6:**
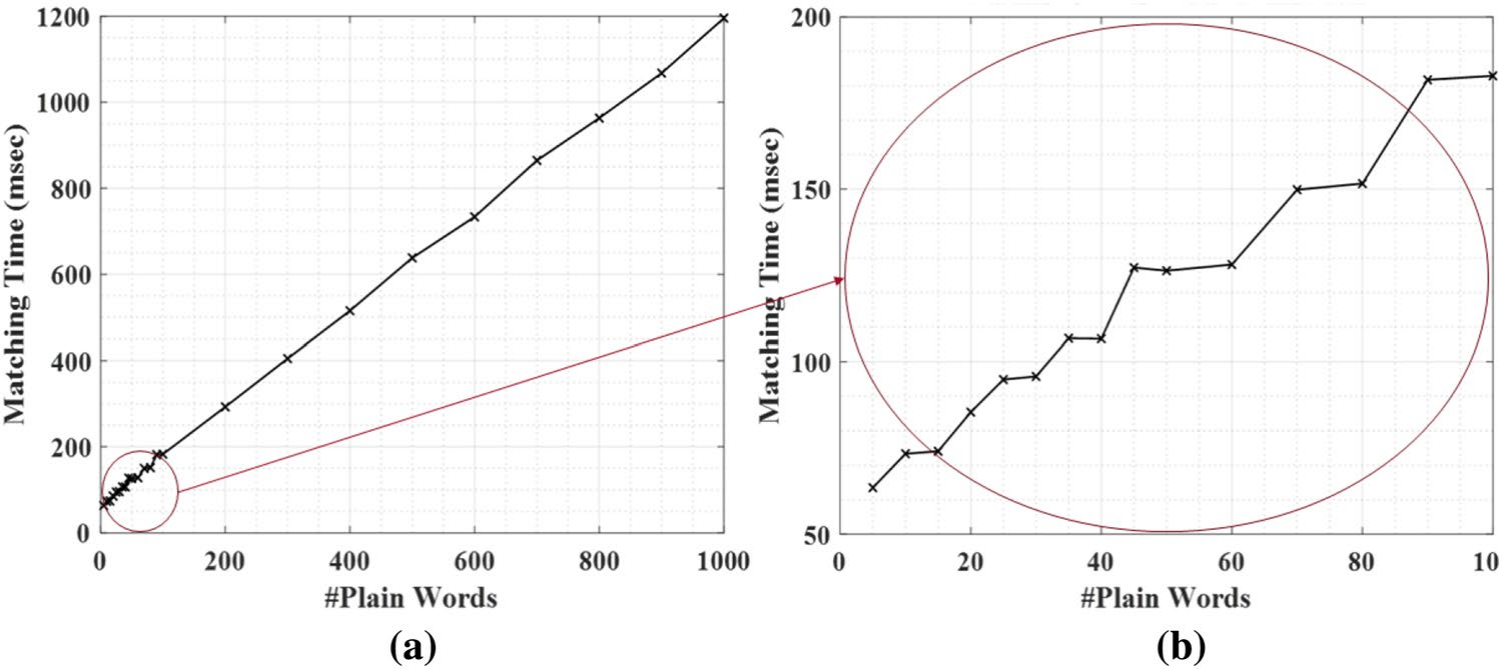
Measured matching time for HDMv1 parallel model implemented with cuFHE against the number of plain words.

**Figure 7:**
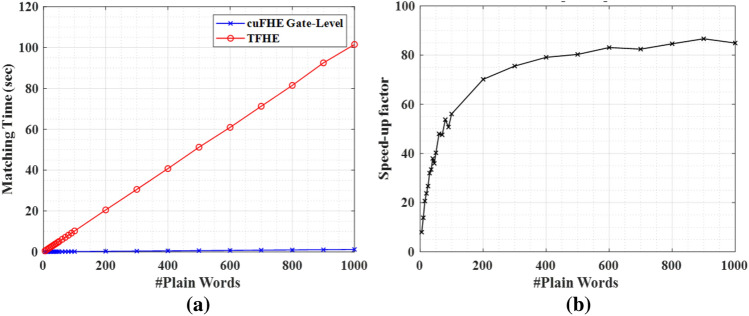
(**a**) Matching time of the HDMv1 parallel model implemented with cuFHE compared to the regular sequential model implemented with TFHE against the number of plain words. (**b**) Speed up gain of using the parallel approach over the sequential one.

Figure 6a shows the measured matching time taken by HDMv1 implemented with the parallel model against the number of plain words. Figure 6b shows a zoomed-in version for the first few values in Fig. 6a which is similar to a staircase. The staircase behavior is reasonable because a linear increase in the number of characters does not imply a linear increase in the number of execution levels which is obvious especially in small increases. Figure 7a shows the matching time of the HDMv1 parallel model implemented with cuFHE and executed on GPU compared to the regular sequential model implemented with TFHE and executed on CPU against the number of plain words. Figure 7b shows the speedup gain of using the parallel model compared to the sequential one. It can be seen that increasing the number of plain characters increases the speed gain till it saturates at about 600 words with a speedup gain of about 83. This saturation gain is close to the theoretical expected limit defined by the number of SMs in the GPU i.e. the maximum number of concurrently executed gates.

**Table 11:** Estimated and measured execution time for hybrid matching of 5, 10, and 50 chars against 1 char cipher rule using the gate-level parallelization implementation.

Platform	#Gates per Level			# Execution levels ($\lceil$#Gates per level / 82$\rceil$)		
	CPU(TFHE)			GPU(cuFHE)		
# Plain Words	5	10	50	5	10	50
L1(HHE_XNOR)	40	80	400	1	1	5
L21(HE_AND)	20	40	200	1	1	3
L22(HE_AND)	10	20	100	1	1	2
L23(HE_AND)	5	10	50	1	1	1
L31(HE_OR)	2	5	25	1	1	1
L32(HE_OR)	1	2	12	1	1	1
L33(HE_OR)	1	1	6	1	1	1
L34(HE_OR)		1	3		1	1
L35(HE_OR)			2			1
L36(HE_OR)			1			1
Total Number (Gates - Execution Levels)	39	79	399	6	7	12
Matching Time Estimation Formula	#Gates * 13 msec			# Execution Levels * 11 msec		
Estimated Matching Time (ms)	507	1027	5187	66	77	132
Measured Matching Time (ms)	506.4	1016.54	5086.48	63.49	73.35	126.34

### Extension to complete rule sets

A certain SURICATA rule^40^ may take the form (**alert tcp 192.168.20.15 20-25 -> 122.234.0.0/16 !17 **), the implementation of such rule using the proposed matching machines require the following:

- Exact match for the protocol “tcp”, which can be done by HDMv1 or HDMv2 (one-byte rule against one-byte plaintext).
- Exact match for the source IP address, which can be done by a 4 HDMv1 or HDMv2 (one-byte rule against one-byte plaintext each) with their results aggregated using 3 HE AND gates
- Range matching for the source port, which can be done by HRMv1, HRMv2, or HRMv3
- Exact match for the first 2 octets of the destination IP address, which can be done by 2 HDMv1 or HDMv2 (one-byte rule against one-byte plaintext each) with their results aggregated using HE AND gate.
- A negated exact match for the destination IP port, which can be done using a HRMv1-e or HRMv3-e followed by HE NOT gate, or by HRMv2.

The results from each matching section are then combined using HE AND gates. If the fastest implementation is required, then HDMv2 and HRMv2 are used when possible. In this case, the rule mentioned above will require a calculation time of: 7 HDMv2 machines (1-byte rule against 1-byte of plaintext) + 1 HRMv2−r + 1 HRMv2−e + 8 HE AND gates + 1 HE NOT gate (Not required in case of using HRMv2 for destination port). The matching time would be mostly consumed by the 8 HE AND gates = 104 ms, since the HDMv2 and HRMv2 adopt look-up table approach so they take insignificant time for matching 1 byte rule against 1 byte plaintext. The memory required by the rule will be 7*634 kB + 2*158.5 MB = 321.3 MB. Of course, speed can be improved by parallization techniques as discussed in section “Performance acceleration with parallel processing”. If saving resources is more important than speed, then HDMv1 and HRMv1 can be used. In this case, the matching time will be 7*91 +790 + 192 + 8*13 = 1723 msec and the memory required will be 7*19.81+237.75+158.5 = 534.92 KB.

The resources required for implementing a complete rule set can be computed while taking into consideration the following parameters:

- **Rule Set**: The rule set properties, including the number of rules and their structure.
- **HE Scheme**: Different HE schemes show different time and memory performance, security, and computation requirements.
- **Matching scheme**: As illustrated in sections “Hybrid direct matching (HDM) algorithms” and “Hybrid range matching (HRM) algorithms”, different matching techniques require different resources.

By measuring the elementary hybrid/HE operations time and memory requirements for a specific HE scheme on a specific machine, like in Tables 9 and 10, the analysis of time and memory requirements described above for a typical rule can be extended to estimate the time and memory requirements for a complete set of rules. In the case of a single processor, single thread, and completely independent rules the computation time of matching a single packet against the whole rule set will be simply the addition of the time required by each rule and the same for memory requirements. However, the modularity of the hybrid gates approach allows for sharing rule segments between multiple rules, e.g., multiple rules have the same source IP address, which effectively reduces the time and memory requirements since this rule segment will be stored once and executed once per packet. Also, the parallelization capabilities of a cloud side matching computer, e.g., parallel CPUs and threads and utilization of GPUs, can effectively reduce the time requirements for matching against the rule set since rules will be matched in parallel. This analysis technique can be used to calculate the total throughput of the complete rule set matching machine and define the limiting network traffic that can be processed through the ONIDS.

## Discussion

GGHLM provides an elegant solution from the mathematical perspective to the problem of executing encrypted NFA on plaintext input. However, it is not suitable for every situation because of multiple issues that are handled better in our approaches.

1. **HE Scheme Limits**: Our approach is library independent, which gives flexibility in choosing the HE library and scheme that matches the requirements. This feature showed great advantage in section “Performance acceleration with parallel processing” where an already existing GPU-accelerated HE library is utilized to build a parallel-processing model that gave additional $\times$ 83 speed gain without having to build a new library from scratch to work with our model as the case in GGHLM. More importantly, our approach can be implemented on top of a bootstrapable library like TFHE which means it will have an infinite depth that allows building any arbitrary circuit. On the other hand, GGHLM adopts a leveled HE scheme, which means it has limited multiplication depth i.e. it can process a limited number of plaintext symbols before decryption becomes mandatory for recovering the data.
2. **Security Level**: The security of our approach depends on the security of the underlying HE library, and the adopted libraries and schemes ensure a minimum of 128 bits of security for our implementations. While changing the configuration parameters in GGHLM should theoretically achieve a security level of 113 bits, these parameters are not the currently available in their implementation^54^ and the available ones allow only for 51 bits of security which may be not suitable for the requirements of some nowadays applications. Moreover, changing the parameters to get a higher security level increases the memory requirements and processing time significantly.
3. **Rule Encryption Time**: In all of our approaches, the maximum rules encryption time is 1.3 s for HRMv2 while encrypting $2^{16}$ possible answers, and all other variations are much less in the msec range. On the other hand, the minimum time required by GGHLM for encrypting the transition matrices for two symbols only in GGHLM-B is 10 s. It also goes up to 22 mins in the case of the HDM task as in Table 9 and reaches extreme results in the case of GGHLM-H16 implementation. The fast rule encryption time is an important factor in certain applications that require frequent rules updates like adaptive NIDSs.
4. **Encrypted Rule Size**: The encrypted rule size has a direct effect on the data transmission costs between the Trusted Client and the cloud. While the encrypted rule size maxes out to 159 MB in our HRMv2, goes to few MBs for HRMv3, and becomes in the KBs range for the others, it starts with 66 MBs for GGHLM-B and goes up to nearly a dozen GBs for the direct matching, which makes the transmission expensive and maybe impractical in some cases.
5. **Maximum Resident Memory**: This indicates how much memory is required to run the algorithm, and it is important to keep this number low in case multiple instances of the algorithm are required to run in parallel, which is always the case with NIDSs that matches against many rules, maybe thousands, in the same time. Our approach has 3 major memory components: (1) The key (fixed), (2) The encrypted rule, and (3) The algorithm implementation and library requirements. The key has a fixed size and can be shared between all algorithm instances so it does not have a significant effect. The encrypted rule size is small in all variations except for HRMv2. A closer look at the table data, it can be noticed that the memory consumed by the library requirements and algorithms, estimated by subtracting the key size and rule size from the maximum resident memory, is almost constant in all variations around 140 MBs. In GGHLM, the memory requirements increase linearly with the, already huge, rule size, giving a maximum resident memory of 14 GBs for the direct matching applications which makes it unpractical to run multiple instances at the same time.
6. **Cloud Matching Time**: The cloud matching time is one of the most important factors as it significantly affects the NIDS’s maximum throughput. HRMv2 reaches an outstanding speed of 1.25 usecs which is $10^5$ times faster than the fastest of all other variations, including GGHLM implementations. It should be noted also that HRMv2 shares with GGHLM an interesting feature, which is the time is completely independent of the operation type, which means it does not leak timing data compared to other HRM variations that show time dependency on the comparison operation type. However, it should be noted that this time dependency does not leak any info about the secret key because it basically does not exist in the cloud at all.
7. **Resources Trade Flexibility**: An NIDS can be set up in different environments with different resources and requirements that range from huge mainframes with vast resources to mobile IoT devices with limited power, and from adaptive environments with high data throughput to light sensor networks with much lower data rate. Hence, flexibility in the resources requirements and computation time would be a strong asset in any proposed NIDS scheme. As can be inferred from Tables 9 and 10, our approach presents different variations for the same task. For instance, HRM variations give HRMv1 option with a 158.5 KB encrypted rule and a searching speed of 386 ms for G operation, and give the HRMv2 option with a 158.5 MB encrypted rule and a searching speed of 1.25 us for the same operation, while also giving HRMv3 that gives in between performance.
8. **Decryption time**: Decryption time is an important timing factor for adaptive NIDSs that update their rules and defense strategies based on feedback results from the cloud. Our approach returns only one encrypted bit to the cloud compared to an encrypted state vector in GGHLM. Built on top of TFHE, our approach achieves a 1 usec decryption time that is more than $10^5$ faster than GGHLM.
9. **Result Size**: The result size of any of the proposed matching algorithms is the size of an encrypted HE bit, which depends directly on the HE scheme. In the case of TFHE, the size of an encrypted bit is 2536 Bytes as shown in Table 8. This size seems to contradict MR3 at first glance; however, the result size is fixed and does not change with the plaintext size. This means the proposed scheme would satisfy MR3 when the rules are used with larg plaintext corpus (when the plaintext size is larger than $\sim$2.4 KB) which agrees with the natural use of an oblivious NIDS idea that performs results batching and does not send the results too frequently to the Trusted Client as demonstrated in section “Extension to complete rules and batching”.
10. **Modular Structure**: An NIDS rule can have multiple parts e.g. a constraint on communication protocol, IP range, and Port range. With our approach, it is straightforward to implement a matching machine for each part of the rule, do the matching, and then combine the results together using HE gates. It is also easy to update one part of the rule without touching the other parts, e.g. update the IP range with the same port range, due to this modular structure. This is not the case with GGHLM, where there is no obvious way to homomorphically combine the state vectors of different NFAs acting on different parts of the rule.
11. **Limitations**: Since nothing comes for free, our superior performance in almost all aspects of the oblivious NIDS system comes at one limitation with respect to the GGHLM. The goal of GGHLM is to protect both the rules data as well as the evaluation circuits as if it is an HE system built on top of a circuit obfuscator. On the other hand, our solution protects only the rules data, not the evaluation circuit, which is more aligned with the goal of homomorphic circuits. Although the adversary cannot identify the type of multiplexer hybrid gates used, which lies on the border between the network plaintext stream and the homomorphically encrypted domain, the number of gates and the structure of the pure HE gates inside the homomorphic domain are visible to the adversary. We believe that this limitation does not affect the operation of the oblivious NIDS under the Hosnest-but-Curios model where we don’t aim at hiding the reality of a network server being an NIDS system, but aim at only protecting the signature rules used within. All modern NIDSs have similar evaluating circuits and the reality of the NIDS is assumed to be known to the adversary.

Hence, we conclude that any rule that must be implemented as a sequential FSM (e.g., complex Regex statements), where the rule data is embedded in the circuit structure itself, is better approached by the GGHLM method. However, any rule that can be implemented as a combinational logic circuit (e.g., ranges and direct matching over any field in the packet header: IP addresses, port numbers, and protocol types, and any direct matching in the packet payload) is much better approached by our method.

## Conclusion

This paper introduced the hybrid logic gates concept along with their multiplexer implementation at the bit level. The implementation methodology guarantees the privacy of the process result and logic operation type, which helps hide the circuit structure. The paper also presented two hybrid matching algorithms using the hybrid binary gates: Hybrid Direct Matching and Hybrid Range Matching, which could be implemented at the core of a privacy-preserving NIDS. The abstract dependence of the proposed approach on the HE libraries allowed testing the algorithms with different libraries and taking advantage of a GPU-accelerated library for building and executing a parallel model to one of the algorithms. The benchmarking results on CPU showed a wide variety of speed $\times$ memory trade in both proposed algorithms variations. A rule encryption time as low as 0.012% compared to the rule encryption time of the state of the art is achieved. The results also showed that the encrypted rule size in our algorithms can be only 0.047% of its similar in the state of the art in, while the matching speed can be 20,000 times faster than the state of the art in some cases. The implemented parallel model on the GPU demonstrates that the matching operations can be made faster with an additional factor of 83 compared to the CPU implementations.

## Data Availability

All data generated or analysed during this study are included in this published article
